# Epigenome Mapping in Quiescent Cells Reveals a Key Role for H3K4me3 in Regulation of RNA Polymerase II Activity

**DOI:** 10.3390/epigenomes8040039

**Published:** 2024-10-22

**Authors:** Shengyuan Zeng, Karl Ekwall

**Affiliations:** Department of Medicine Huddinge, Division of Biosciences and Nutrition, Karolinska Institute, NEO Building, SE-141-83 Huddinge, Sweden; shengyuan.zeng@ki.se

**Keywords:** cellular quiescence, G_0_ arrest, fission yeast, histone modifications, H3K4me3, Set1C/COMPASS, RNA polymerase II, regulation of gene expression

## Abstract

(1) Background: Quiescent cells are those that have stopped dividing and show strongly reduced levels of gene expression during dormancy. In response to appropriate signals, the cells can wake up and start growing again. Many histone modifications are regulated in quiescence, but their exact functions remain to be determined. (2) Methods: Here, we map the different histone modifications, H3K4me3, H3K9ac, H3K9me2, and H3K9me3, and the histone variant H2A.Z, comparing vegetative and quiescent fission yeast (*S. pombe*) cells. We also map histone H3 as a control and RNA polymerase II (phosphorylated at S2 and S5) to enable comparisons of their occupancies within genes. We use ChIP-seq methodology and several different bioinformatics tools. (3) Results: The histone modification mapping data show that H3K4me3 changes stand out as being the most significant. Changes in occupancy of histone variant H2A.Z were also significant, consistent with earlier studies. Regarding gene expression changes in quiescence, we found that changes in mRNA levels were associated with changes in occupancy of RNA polymerase II (S2 and S5). Analysis of quiescence genes showed that increased H3K4me3 levels and RNA polymerase II occupancy were super-significant in a small set of core quiescence genes that are continuously upregulated during dormancy. We demonstrate that several of these genes were require Set1C/COMPASS activity for their strong induction during quiescence. (4) Conclusions: Our results imply that regulation of gene expression in quiescent cells involves epigenome changes with a key role for H3K4me3 in regulation of RNA polymerase II activity, and that different gene activation mechanisms control early and core quiescence genes. Thus, our data give further insights into important epigenome changes in quiescence using fission yeast as an experimental model.

## 1. Introduction

Cellular quiescence is a reversible differentiation state when cells become dormant. The quiescent cells are changing the gene expression program to reduce metabolic functions and adapt to new cellular environments. The ability of cells to exit from the cell cycle into quiescence and to re-enter the cell cycle from quiescence is crucial for tissue maintenance and repair in multicellular organisms [1]. Moreover, it is an important longevity strategy for unicellular organisms to ensure cell survival during long periods of limited nutrients [2]. Understanding the regulation and maintenance of quiescent cells is also of medical importance, since quiescence can allow for cancer cells to escape treatment [3].

The fission yeast *Schizosaccharomyces pombe* (*S. pombe*) has been a popular model organism for cell division cycle studies for several decades [4]. It is also an excellent model for studies of cellular quiescence because of its highly synchronized and reversible G_0_ arrest upon nitrogen starvation and well-developed methodology for genetics [5,6]. *S. pombe* cells can survive for several weeks in quiescence highly synchronized in the G_0_ stage of the cell cycle [5]. *S. pombe* is also a suitable model system for studies of epigenetic mechanisms, histone modifications in particular [7].

There are many indications that histone modifications are controlled during cellular quiescence and contribute to the balance between proliferation and dormancy in different cellular models (reviewed in [8]). However, a detailed understanding of the functional role of histone modifications in quiescence entry, maintenance of viability in quiescence, and quiescence exit is currently lacking. In fission yeast, the RNA interference process and the histone H3K9 methyltransferase Clr4 have been found to be essential in quiescent fission yeast cells [9,10]. To deepen our understanding of epigenome changes in quiescent cells, we earlier performed a genetic screening of *S. pombe* that identified additional chromatin-modifying enzymes, including the histone acetyl-transferase complex SAGA, the histone deacetylase complex RPD3, and histone H3K4 methyltransferase complex Set1C/COMPASS, as being essential for viability in quiescence [11]. Thus, it was clear that further studies were required to determine more exactly why the different modifications regulated by these enzymes are required for viability in quiescence. More recently, we mapped gene expression changes in quiescent cells by RNA-seq [12]. Here, here we mapped alterations of the epigenome in quiescent fission yeast cells and correlated these to changes in gene expression. We used antibodies against several different histone modifications, H3K4me3, H3K9ac, H3K9me2, and H3K9me3, and the histone variant H2A.Z, to address their function in quiescence. We also mapped histone H3 as a control and RNA polymerase II (phosphorylated at S2 and S5) to enable comparisons of their occupancies within genes. Our analysis reveals a dramatic change in the epigenome and that H3K4 methylation clearly stands out, showing the most significant histone modification changes in quiescent cells compared to vegetative fission yeast cells.

## 2. Results

### 2.1. A Dramatic Change in the Epigenome of Quiescent Cells

We performed ChIP-seq analysis of several histone modifications, the histone variant H2A.Z, and two phosphorylated forms of RNA pol II in vegetative and quiescent fission yeast cells. Quiescence was induced by shifting the culture media from nitrogen containing minimal medium during vegetative logarithmic growth (at a cell density of 1–5 × 10^6^ cells/mL) to nitrogen-free minimal medium and then incubating for 24 h to obtain the quiescent cells. After chromatin preparation from the vegetative and quiescent cells, we added *Drosophila* spike-in chromatin to allow for detection of global changes in occupancy of the different modifications.

**Figure 1:**
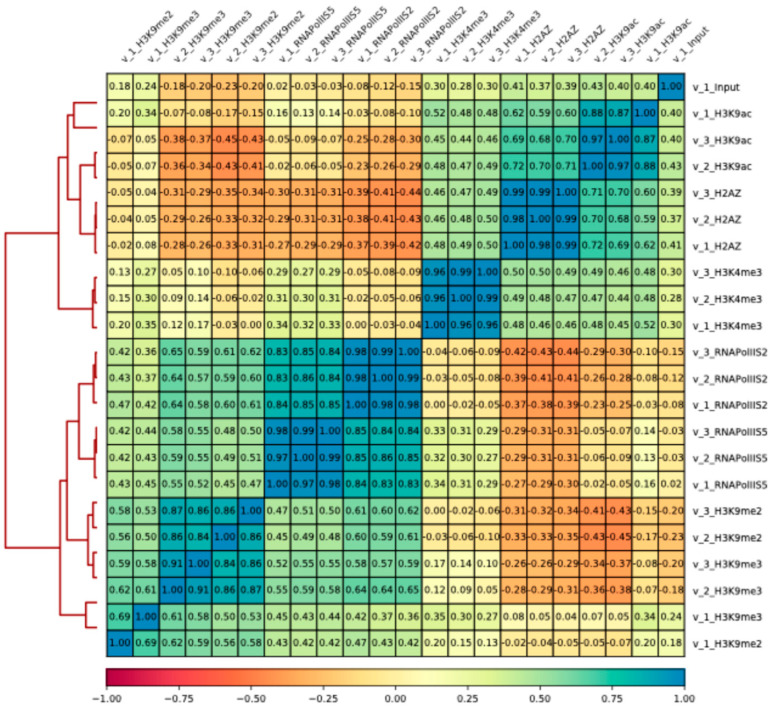
Correlation matrix of vegetative (V) cells. Heat map showing pairwise Spearman correlation coefficients for all vegetative ChIP-seq samples calculated based on ranks of each bin.

**Figure 2:**
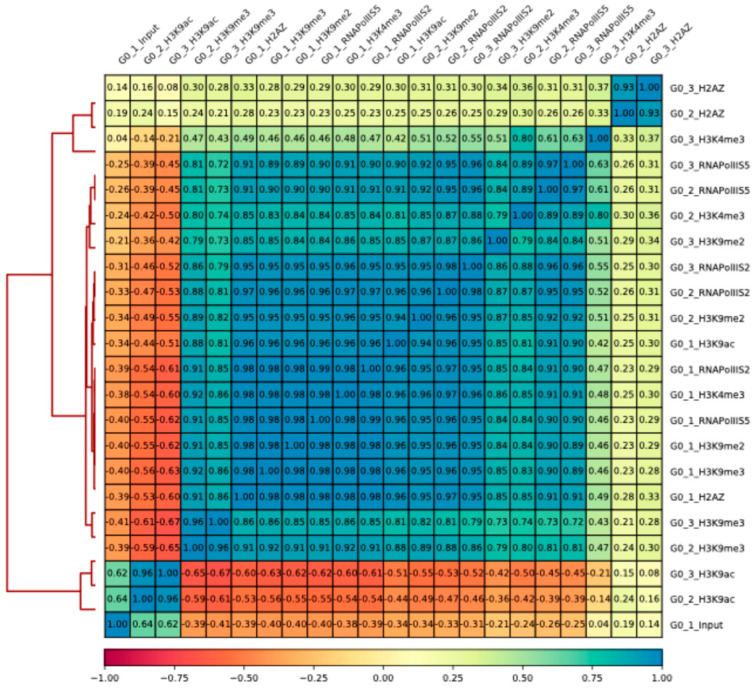
Correlation matrix of quiescent (Q) cells. Heat map showing pairwise Spearman correlation coefficients for all quiescent ChIP-seq samples calculated based on ranks of each bin.

First, the ChIP-seq data were analyzed, including biological triplicates for each antibody, using a global scale clustering approach. In vegetative (V) cells there were clear correlations and anti-correlations between groups of histone modifications, and H2A.Z (detected by the Myc epitope-tag). For example, the heterochromatin marks H3K9me2 and H3K9me3 were anti-correlated with the euchromatic marks H3K4me3, H3K9ac, and H2A.Z, as expected (Figure 1). There was also a strong correlation between the two RNA polymerase II epitopes (S2 and S5). In quiescent (Q) cells, we did not observe any clear correlations or anti-correlations in the heatmap (Figure 2). Thus, the overall impression is that the epigenome at a global scale becomes much less organized in Q cells compared to V cells.

### 2.2. Changes in Genes in TSS and ORF Regions

**Figure 3:**
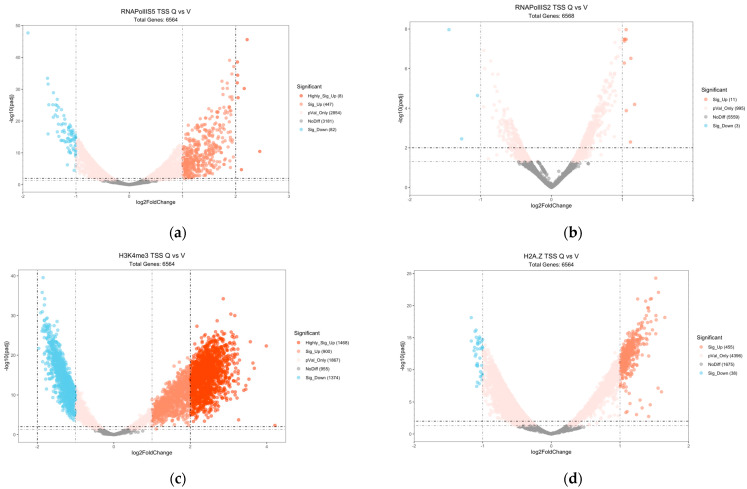

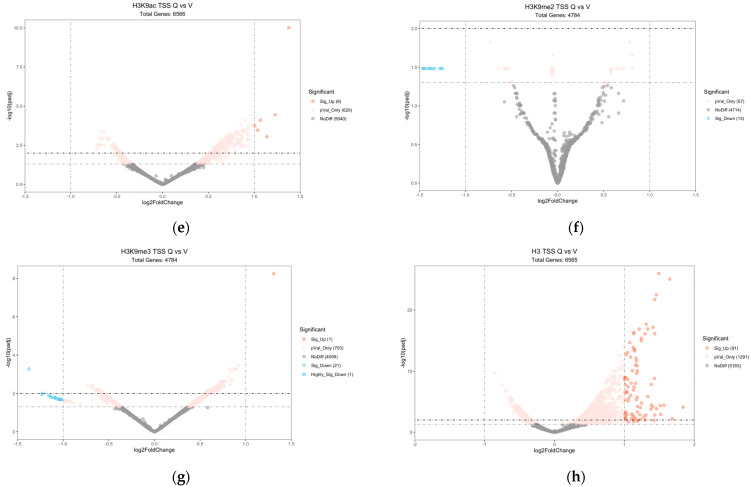
Volcano plots. Batch effects were removed using DEseq2 (see Section 4). Significantly changed TSS regions in Q cells vs. V cells, are labelled by red, pink, light-blue, and blue colors. Highly significant up: log fold change > 2.0; *p* < 0.01. Significant up: log fold change > 1.0; *p* < 0.05; *p*-value only: log fold change < 1.0 or >−1.0; *p* value < 0.05. Highly significant down: log fold change < −2.0; *p* < 0.01. Significant down: log fold change < −1.0; *p* < 0.05. No difference *p* > 0.05. Panels (**a**–**h**) show the different ChIP-seq signals comparing Q cells v.s V cells: (**a**) RNA Pol II S5; (**b**) RNA pol II S2; (**c**) H3K4me3; (**d**) H2A.Z; (**e**) H3K9ac; (**f**) H3K9me2; (**g**) H3K9me3; (**h**) H3.

Next, we analyzed transcription start site (TSS) regions and open reading frame (ORF) regions separately, comparing Q and V cells using volcano plots. For TSS regions, we used a 400-base-pair window size of 200 base pairs upstream and 200 downstream of the TSS, and for ORF regions, we used the entire ORF region (from TSS to TES plus and minus 200 base pairs). Again, the triplicate samples were used for each antibody; however, batch effects were removed to allow for comparisons of fold changes and statistical significance. This analysis revealed that the most significant changes in TSS regions in Q cells compared to V cells (Q vs. V) were histone methylation levels at H3K4me3 and occupancy of the histone variant H2A.Z (Figure 3c,d). Changes in the other mapped modifications were much less significant in Q cells (Figure 3e–h). The results showed that as many as 1468 genes had a super-significant, and 900 genes had a significant increase in H3K4me3 levels, whereas 1374 genes had significantly reduced H3K4me3 levels in TSS regions of Q cells (Figure 3c). Regarding H2A.Z, 455 genes had significantly increased levels, and 38 genes had significantly reduced levels in TSS regions of Q cells (Figure 3d). A smaller number of genes showed TSS changes in RNA polymerase II occupancies in Q cells (Figure 3a,b). Only 8 genes showed a super-significant increase, 447 genes showed a significant increase, and 38 genes showed reduced RNA polymerase II occupancy phosphorylated at S5 in TSS regions (Figure 3a). An even lower number of genes show significant changes in RNA polymerase II occupancy phosphorylated at S2 in TSS regions of Q cells (Figure 3b).

The most significant changes in ORF regions of Q cells were RNA polymerase II CTD phosphorylated at S2, S5, and H3K4me3 (Supplementary Figure S1). A small number of genes also showed significantly reduced H3K9me2 or -me3 levels in ORF regions in Q cells.

### 2.3. Gene Expression Changes Compared to Epigenome Changes

**Figure 4:**
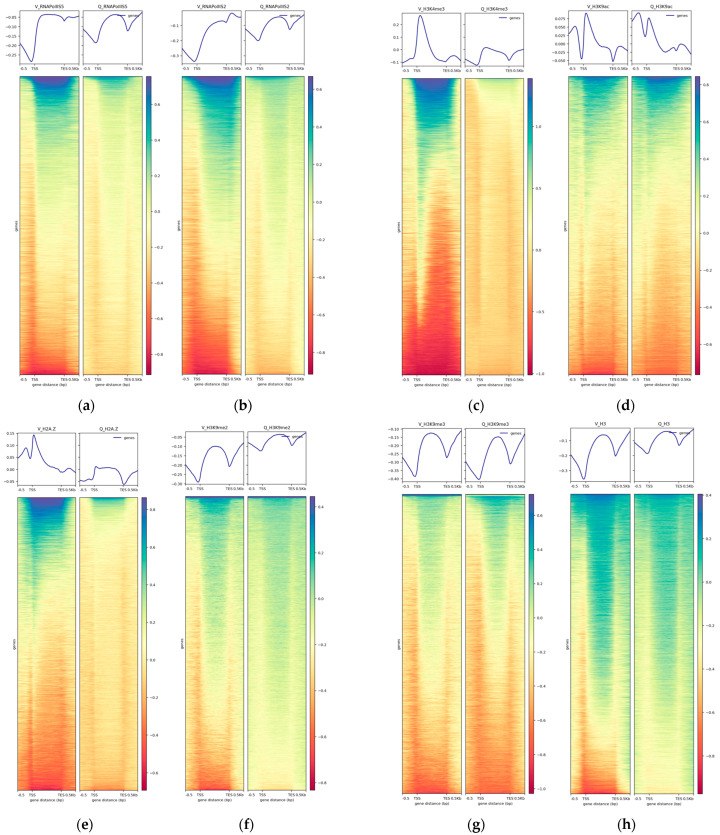
TSS to TES region heatmaps. Sequencing depth and input normalized ChIP-seq signals (read counts). The color gradient from blue to red indicates high to low signals in the different regions. The plot above each heatmap displays the average signal in the different regions. The panels show the ChIP-seq signals in V cells and Q cells as indicated (**a**) RNA Pol II S5; (**b**) RNA pol II S2; (**c**) H3K4me3; (**d**) H3K9ac; (**e**) H2A.Z; (**f**) H3K9me2; (**g**) H3K9me3; (**h**) H3.

To gain a more detailed understanding of the epigenomic changes, we investigated the average gene profiles in Q cells compared to V cells using TSS to TES (transcription exit site) region heat maps. This analysis shows the aggregated occupancy tendencies for each ChIP-seq map (including triplicates) and displays the average profile for all 6612 genes in the fission yeast genome (Figure 4). RNA polymerase II phosphorylated at S5 showed a typical TSS profile with a TSS peak in V cells that was much less pronounced in Q cells (Figure 4a). RNA polymerase II phosphorylated at S2 showed a typical TSS profile with a TES peak in V cells that was much less pronounced in Q cells (Figure 4b). Regarding the mapped histone modifications, there was a clear TSS peak of H3K4me3 in V cells, but this peak was nearly abolished in Q cells (Figure 4c). Similarly, a peak of H2A.Z was seen at the TSS of V cells, but it was less evident in Q cells (Figure 4e). Again, H3K9me2, -me3, and -ac marks did not show any obvious changes between Q and V cells in this analysis, and the H3K9me2 and -me3 signals were very similar to the control histone H3 signals (Figure 4d,f–h).

**Figure 5:**
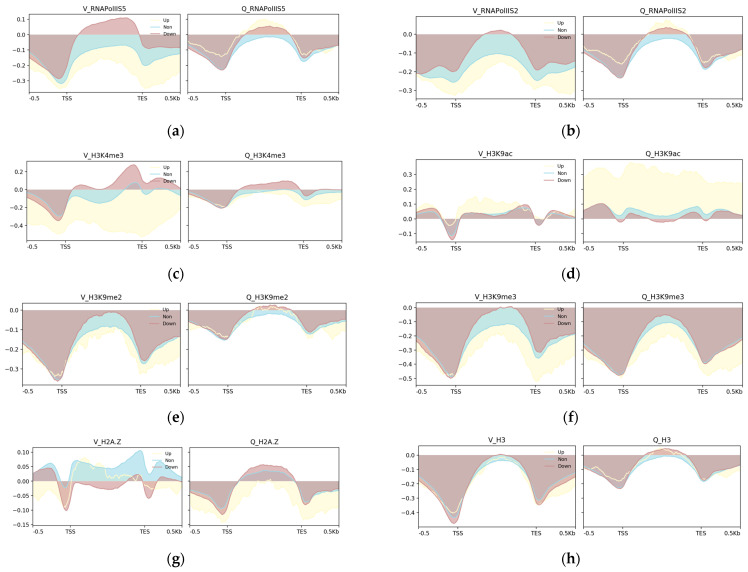
Differential TSS-to-TES profile plots. Sequencing depth and input normalized. Plot profiles for upregulated (yellow area), downregulated (brown area), and non-changed genes (blue area) in Q vs. V cells for the different ChIP-seq signals (read counts) as indicated. ‘Up’ = upregulated in Q cells; ‘Non’ = non-changed in Q cells; ‘Down’ = downregulated in Q cells. The panels show the average ChIP-seq signals in V cells and Q cells as indicated (**a**) RNA Pol II S5; (**b**) RNA pol II S2; (**c**) H3K4me3; (**d**) H3K9ac; (**e**) H3K9me2; (**f**) H3K9me3; (**g**) H2A.Z; (**h**) H3.

Next, we made differential TSS-to-TES profile plots for upregulated, non-changed, and downregulated genes to investigate the epigenome vs. gene expression tendencies at in Q cells compared to V cells (Figure 5). For this purpose, we used our previously published RNA-seq data as a measurement of gene expression, i.e., spike-in-normalized mRNA levels [12]. This dataset contains 149 upregulated genes, 5255 non-changed genes, and 1208 downregulated genes in Q cells after 24 h of nitrogen starvation compared to V cells.

As expected, the upregulated genes in Q cells had lower RNA polymerase II S5 and S2 levels in V cells compared to Q cells, whereas the downregulated genes had higher RNA polymerase II S5 levels in V cells (Figure 5a,b). Regarding the histone modifications, H3K9me2 and -me3 behaved similarly to the control histone H3, showing only small changes in the gene profiles between Q and V cells (Figure 5e,f,h). In sharp contrast, H3K4me3, H3K9ac, and H2A.Z showed dramatic changes between Q and V cells (Figure 5c,d,g). This indicated that the latter epigenome marks are linked to changes in gene expression in Q cells compared to V cells.

### 2.4. Chromatin State Changes vs. Gene Expression Changes in Quiescent Cells

**Figure 6:**
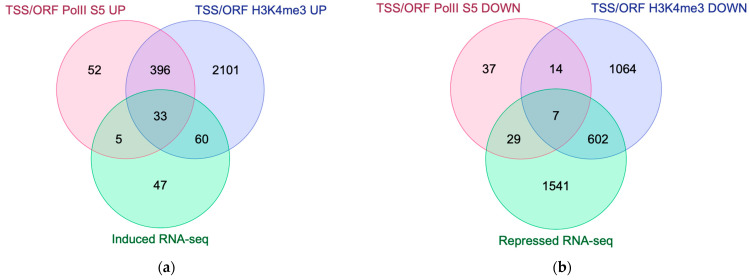
Venn diagram. Chromatin state changes comparing RNA Pol II S5 and H3K4me3 (ChIP-seq signals) vs. gene expression changes (RNA-seq). Panel (**a**) shows the number of upregulated (induced) genes in Q cells that change to an active chromatin state and panel (**b**) shows the number of downregulated genes in Q cells that change to a repressed chromatin state.

Next, we identified regulated genes that change their chromatin state in Q cells vs. V cells by comparing with spike-in-normalized RNA-seq data (from Reference [12]). We compared lists of upregulated and downregulated genes in Q cells with lists of IGR and ORF regions that were significantly changed (up or down) in our chromatin analysis (Figure 3 and Figure S1). We used H3K4me3 and RNA pol II S5 occupancies in TSS or ORF regions as markers for the active chromatin state. These comparisons revealed a set of 33 genes showing a change to an active state, and a smaller set of seven genes that lost the active state in Q cells (Figure 6; Table 1).

The Gene Ontology (GO) terms indicated that many of the upregulated genes that change to an active chromatin state in Q cells encode metabolic enzymes, membrane transporters, and non-coding RNA of unknown function (Table 1). The downregulated genes identified by this approach include the genes for histone H3 (*hht1^+^*) and the 60S ribosomal protein (*rpl27^+^*). The upregulated genes include the *gdp3^+^* gene encoding a glyceraldehyde dehydrogenase and three genes from same chromosomal region: SPBPB21E7.02c, SPBPB21E7.10, and SPBPB21E7.11.

**Figure 7:**
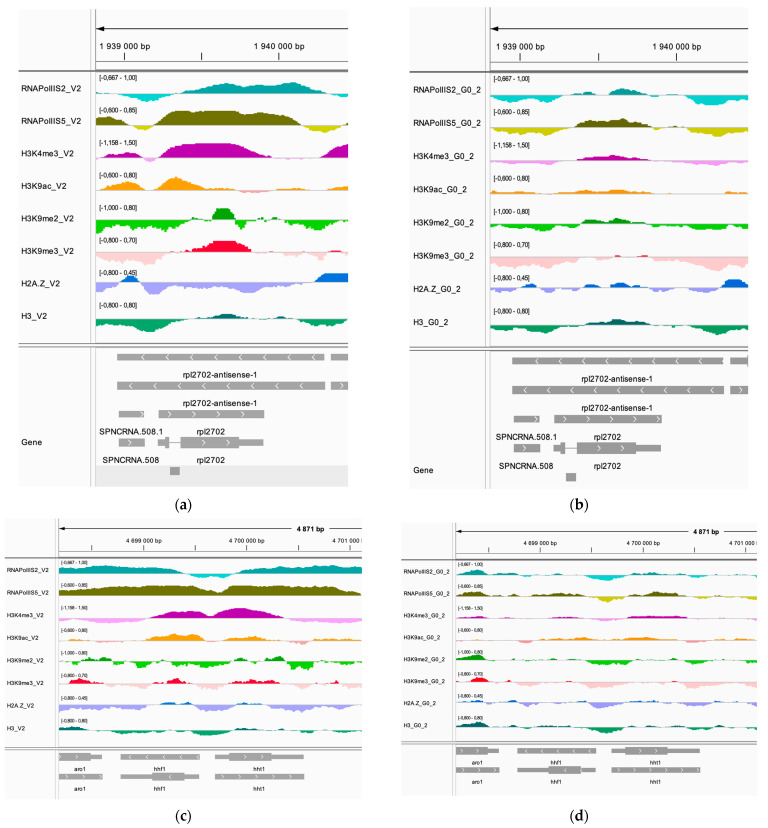

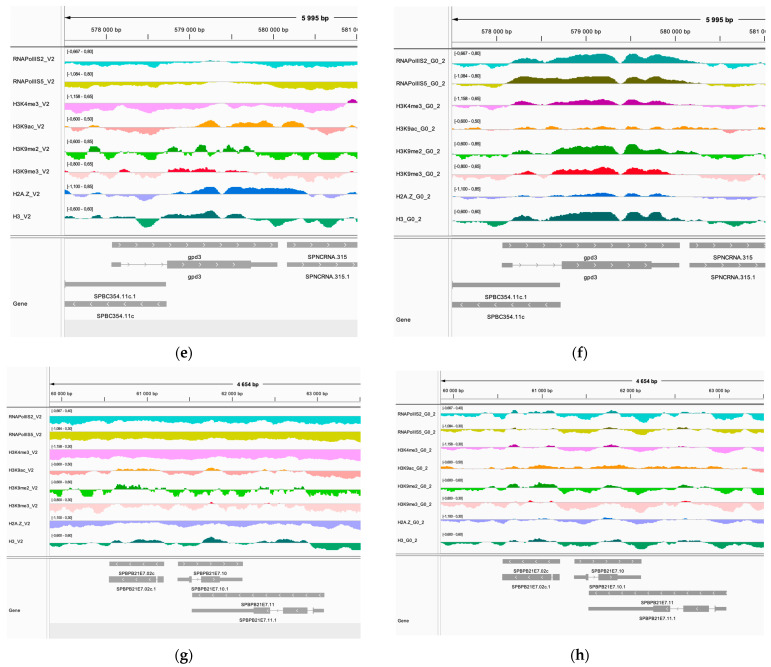
Integrative Genomics Viewer (IGV) displays showing examples of genes changing their chromatin state in Q cells. The first panel shows the *rpl27^+^* gene region in V cells (**a**) and Q cells (**b**). The second panel shows the *hhf1^+^* and *hht1^+^* gene region in V cells (**c**) and Q cells (**d**). The third panel shows the *gdp3^+^* gene region in V cells (**e**) and Q cells (**f**). The fourth panel shows the *SPBPB21E7* region containing three genes in V cells (**g**) and Q cells (**h**). ChIP-seq signals for RNA Pol II S2, RNA Pol II S5, H3K4me3, H3K9ac, H3K9me2, H3K9me3, H2A-Z, and H3 are indicated in each panel.

Integrated Genomics Viewer (IGV) images of these genes are shown in Figure 7 and Supplementary Figure S2. It was evident for genes that become repressed (*rpl27^+^* and *hht1^+^*) that the active histone marks H3K4me3 and H3K9ac and occupancies of RNA Pol II S2 and RNA Pol II S5 were much reduced in Q cells compared to V cells (Figure 7a–d). Conversely, the genes that become active in Q cells had gained the active histone marks H3K4me3 and H3K9ac and showed increased occupancies of RNA Pol II S2 and RNA Pol II S5, whereas the histone H3 control, H2A.Z, and the repressive H3K9me2 and H3H9me3 marks were relatively unchanged at these genes (Figure 7d–g). Hence, the IGV displays support changes in chromatin states for affected genes listed in Table 1.

### 2.5. Epigenome Changes at Upregulated Core Quiescence Genes

**Figure 8:**
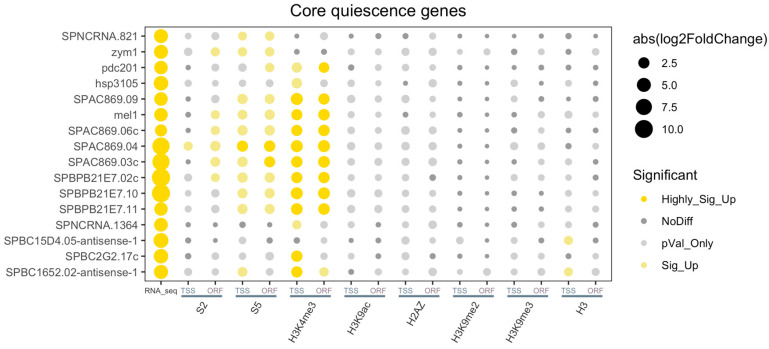
The majority of core quiescence genes show a significant increase in H3K4me3 and RNA polymerase S5. A bubble plot of core quiescence genes generated by Deseq2 tool is shown. RNA_seq = gene expression levels measured by RNA-seq; S2 = RNA polymerase II S2; S5 = RNA polymerase S5; and histone marks, as indicated in TSS and ORF regions, respectively. The gene names are indicated in the left margin.

**Figure 9:**
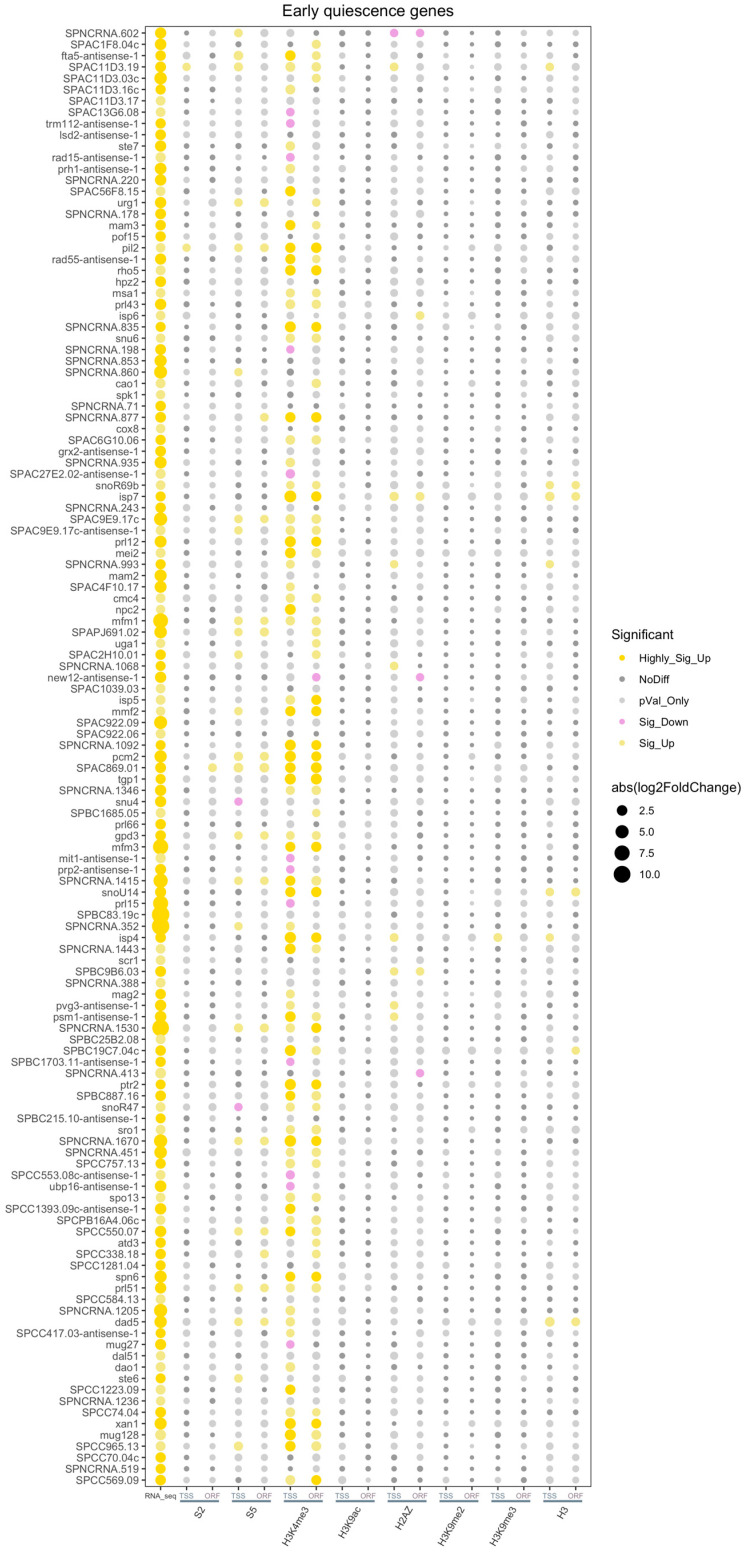
A small subset of early quiescence genes shows a significant increase of RNA polymerase II S5 and H3K4me3. A bubble plot of core quiescence genes generated by Deseq2 tool is shown. RNA_seq = gene expression levels measured by RNA-seq; S2 = RNA polymerase II S2; S5 = RNA polymerase S5; and histone marks, as indicated in TSS and ORF regions, respectively. The gene names are indicated in the left margin.

Our previous study showed that 16 of the 149 early upregulated genes at 24 h remained upregulated even after 1 week and 2 weeks in quiescence [12]. Based on their pattern of continuous upregulation gene expression in Q cells these 16 genes were defined as ‘core quiescence genes’. To investigate if there are any epigenome changes linked to these specific categories of early upregulated and core quiescence genes, we performed more detailed analyses using ‘bubble’ plots and ‘pie’ diagrams (Figure 8, Figure 9 and Supplementary Figures S3 and S4; Supplementary Table S1). For this analysis, we also used the same window size for TSS and ORF regions as for the volcano plots (See above).

The bubble plots revealed highly significant increases of RNA polymerase II S5 and H3K4me3 levels in TSS and ORF regions of many of the core quiescence genes (Figure 8). In contrast, only a fraction of the early upregulated quiescence genes showed a significant increase of these two epigenome features (Figure 9). The pie charts confirmed that a large proportion, 68.7% of core quiescence genes, had increased RNA polymerase II S5 levels in the TSS regions compared to only 18.6% of early quiescence genes (Supplementary Figures S3 and S4). These findings indicate that most of the early upregulated mRNAs are post-transcriptionally stabilized in Q cells compared to V cells, whereas mRNAs of most of the core quiescence genes are actively transcribed by RNA polymerase II in Q cells. Consistent with this notion, the H3K4me3 levels are significantly increased in 81.3% of TSS regions at core quiescence genes compared to 54.3% at early genes (Supplementary Figure S3). Apart from H3K4me3, the other investigated histone marks did not show significant changes at core quiescence genes in Q cells (Figure 8), whereas a few of the early genes also showed significant changes in H2A.Z levels (Figure 9).

Thus, this analysis revealed that most of the early upregulated quiescence genes are post-transcriptionally regulated, i.e., their mRNA is stabilized in Q cells compared to V cells without recruitment of RNA polymerase II. The increase in H3K4me3 observed in TSS regions of 54,3% of these early upregulated genes may be a memory of transcription that occurred before 24 h of nitrogen starvation. In contrast, the core quiescence genes show new recruitment of RNA polymerase II to their TSS regions indicating that continuous transcription of these genes occurs in Q cells. Also, the induced transcription of core quiescence genes by RNA polymerase II in Q cells seems strongly correlated to an increase of the H3K4me3 levels (see Section 3).

### 2.6. Gene Expression Changes in Core Quiescence Genes in a Set1C/COMPASS Mutant

**Figure 10:**
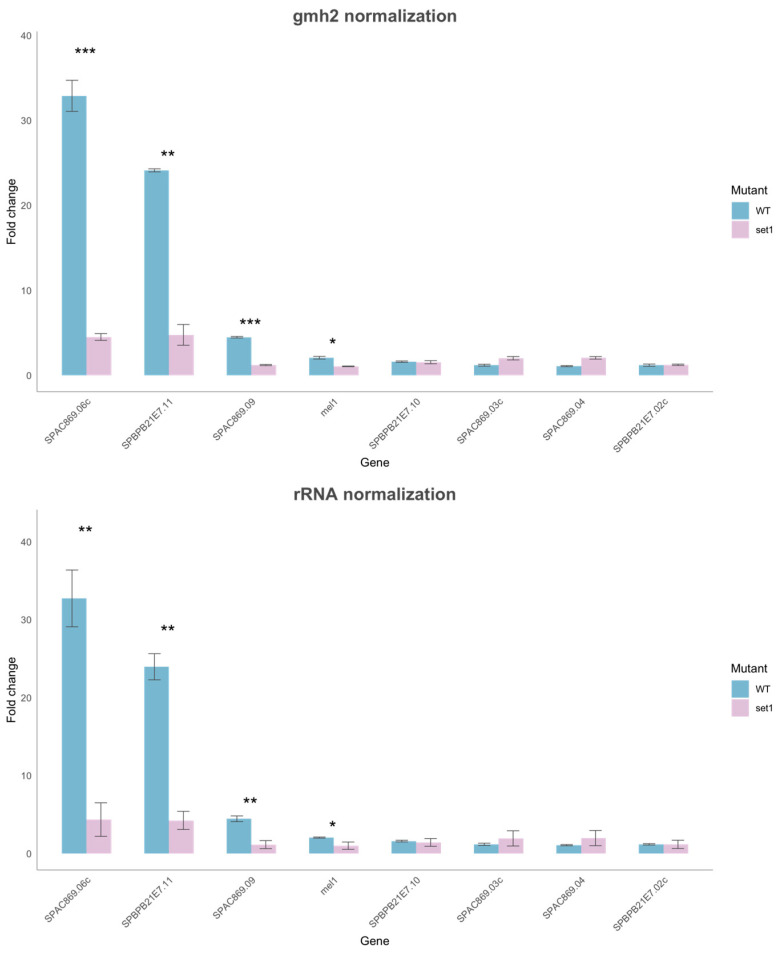
Real-time quantitative PCR (RT-QPCR) analysis of gene expression for core quiescence genes in Q cells vs. V cells. The 2^−ΔΔCT^ method for relative quantitation was used (see Materials and Methods). Statistical significance for reduced expression in *set1Δ* vs. WT is indicated (paired T-test): *p* < 0.001 “***”; *p* < 0.01 “**”; *p* < 0.05 “*”. Panels: (top) *gmh2^+^* normalized data; (bottom) rRNA normalized data.

To investigate if H3K4me3 plays a role in the induction of RNA polymerase II transcription in core quiescence genes, we performed qRT-PCR analysis. We measured the expression of eight subtelomeric core quiescence genes in wild-type cells and *set1Δ* mutant cells in the vegetative state (V cells) and in the quiescent state after 24 h of nitrogen starvation (Q cells). We used the *set1Δ* mutant for this experiment since it is devoid of H3K4 methylation [13]. The eight core quiescence genes are located in the *tel1R* and *tel2L* subtelomeric regions [12]. As control genes, we used the *gmh2*^+^ gene and the rRNA gene since they are not induced in Q cells. We found that the only two of the eight tested genes (SPAC896.06c and SPBPB21E7.11) showed a strong and induction in quiescent WT cells using three biological repeats (Figure 8 and Figure S5). Interestingly, the strong upregulation of both these genes required the *set1^+^* gene, encoding the catalytic subunit of Set1C/COMPASS complex. However, other genes, SPAC869.09 and *mel1^+^*, were moderately induced, and this also required Set1 (Figure 10; Supplementary Figure S5). Thus, we conclude that H3K4 methylation by Set1C is needed at several core quiescence genes for their moderate and strong induction in Q cells.

## 3. Discussion

### 3.1. Strong Changes of the Epigenome in Quiescent Fission Yeast Cells

Here we present genome-wide maps for several different histone modifications, occupancy of the histone variant H2A.Z, and two phosphorylated forms of RNA polymerase II in vegetative (V) and quiescent (Q) cells. The comparison revealed a dramatic change in the epigenome of Q cells. We find that the epigenome of Q cells is much less well organized compared to that of V cells. It is known that Q cells have globally repressed for transcription with total mRNA levels at about 20% per cell compared to V cells [14]. Thus, it is likely that the drastic changes we observe in the epigenome in Q cells reflect the global repression of mRNA transcription. Our analysis of TSS regions showed that many significant epigenome changes between V cells and Q cells are indeed occurring at transcription start sites supporting this notion. At a global level the heatmaps showed that the TSS peak of RNA polymerase II S5 and the TES peak of RNA polymerase II S2 were both less pronounced in Q cells compared to V cells. RNA polymerase II S5 is linked to initiation whereas S2 is coupled to elongation of transcription [14]. The strong changes between Q and V cells confirm that many genes are repressed in Q cells. ChIP-seq analysis confirmed that changes in occupancy of the histone variant H2A.Z are important in regulation of gene expression during quiescence. This result is consistent with as essential role for the chromatin remodeler Ino80C that controls H2A.Z localization for survival of fission yeast cells in quiescence [12].

### 3.2. A Conserved Role for H3K4 Methylation in Quiescent Cells

Our ChIP-seq results also show very strong changes of H3K4me3 in Q cells. We observe both a global reduction in downregulated genes and an increase in H3K4me3 in upregulated genes. H3K4 methylation was previously implicated in quiescence by the lysine-to-alanine point mutation in histone H3 (K4A) in the distantly related budding yeast [15]. The K4A mutant budding yeast cells are devoid of H3K4me and show reduced viability after 5–10 days in quiescence. In addition, mapping of H3K4me3 in logarithmically growing and quiescent budding yeast cells revealed a global reduction in this modification in 7-day-old quiescent cells [15]. Thus, a role for H3K4 methylation in quiescence is shared between the distantly related fission and budding yeasts. It is possible that a function for H3K4 methylation in quiescence evolved early in eukaryotes. Quiescent mouse hematopoietic stem cells (HSCs) show a reduction in H3K4me3 levels [16]. Hence, a key role for H3K4 methylation may also be conserved in quiescent mammalian cells.

We show that 33 of the upregulated quiescence genes identified by RNA-seq [12] change their chromatin state to active, defined by RNA pol II S5 and H3K4me3 signals, whereas only 7 of the downregulated genes lose their active chromatin state. The latter group includes genes encoding a 60S ribosomal protein (*rpl27^+^*) and histone H3 (*hht1^+^*). This is expected for dormant cells arrested in the cell cycle since ribosome biogenesis is reduced, and the *hht1^+^* gene is only expressed in V cells during S phase. The group of 33 upregulated genes that change to an active chromatin state contain genes encode several metabolic enzymes and transmembrane transporters. An upregulation of these cellular functions in Q cells is consistent with earlier observations [2].

### 3.3. Different Mechanisms for Activation for Early and Core Quiescence Genes

We used a timepoint of 24 h of nitrogen starvation, i.e., 1-day-old Q cells, for our mapping studies because it is known that the transcriptome is already strongly reduced at this early timepoint [14]. At the 24 h timepoint, only 149 genes are upregulated, and as many as 1208 genes are downregulated [12]. Of the 149 ‘early’ genes, 16 were also found to be upregulated after 7 days and 2 weeks of quiescence and were defined as ‘core’ quiescence genes [12]. The comparison with RNA polymerase II occupancies revealed that most of the early genes are likely transcribed before entry into quiescence, since S2 and S5 occupancies are relatively low in most of these genes in 1-day-old quiescent cells. In budding yeast, RNA polymerase II occupancy was less correlated to mRNA abundance in 7-day quiescent cells compared to vegetative cells, suggesting that many mRNAs were transcribed in vegetative cells and stored in quiescent budding yeast cells [15]. This notion is consistent with our observations for early quiescence genes in fission yeast.

### 3.4. Core Quiescence Genes Require Set1C Activity for Their Induction in Q Cells

Most of the core quiescence genes in fission yeast displayed significantly increased occupancies of RNA polymerase II phosphorylated at S2 and S5 in their TSS and ORF regions. This suggests that gene activation involving recruitment of RNA polymerase II and transition into elongation mode is occurring in the Q cells at these genes. The core quiescence genes also show a super-significant increase in H3K4me3, suggesting that this modification is mechanistically and temporarily linked to both initiation and elongation steps in the Q cells. Set1 is the only H3K4 methyltransferase enzyme in fission yeast, and therefore the Set1 activity is essential for H3K4me3 methylation [13]. Here we demonstrate that Set1C is needed in core quiescence genes for their moderate and strong induction in Q cells.

### 3.5. A Direct Role for H3K4me3 in Activation of Quiescence Genes?

The textbook view of gene transcription by RNA polymerase II is that S5 phosphorylation of S5 occurs during the initiation step and that S2 is phosphorylated later during the elongation step [17]. Tri-methylation of H3K4 occurs during initiation of transcription as a consequence of S5 phosphorylation, and the role of this histone modification is thought to facilitate several aspects of transcription including recruitment of chromatin remodeling and elongation factors. It was recently demonstrated by acute depletion experiments in mammalian cells that Set1C/COMPASS is required for pause-release of RNA polymerase II and elongation of transcription [18]. Based on this, it was suggested that H3K4me3 stimulates transcription elongation by RNA polymerase II directly by recruitment of an RNA processing factor, called Integrator subunit 11 (INTS11), that is involved in the pause–release of RNA polymerase II in mouse embryonic stem cells [18]. In fission yeast, Set1C is associated with both S5- and S2-phosphorylated forms of RNA polymerase II [13]. There is a conserved homologue of INTS11 in fission yeast called Ysh1 that is involved in mRNA processing [19]. Thus, it is possible that Set1C has a similar function in fission yeast cells.

### 3.6. Quiescence Mortality Phenotypes of Set1C/COMPASS Mutants

Interestingly, our new results are consistent with our previous genetic screening for chromatin regulators required in quiescence, showing that the Set1C/COMPASS enzyme complex is essential for survival in quiescence. The screening-identified genes encoding proteins from several histone modifying complexes, including RPD3 and SAGA, which affect histone acetylation, and Set1C/COMPASS, which is responsible for H3K4 methylation [11]. The Set1C/COMPASS complex contains eight conserved subunits in fission yeast: Ash2 (ASH2L), Sdc1 (DPY30), Set1 (SETD1A or SETD1B), Shg1, Spf1 (CXXC1), Swd1(RBBP5), Swd2 (WDR82), Swd3 (WDR5) (the names of the human protein orthologs are written in capital letters) [20]. We reported that the Set1C gene deletion mutants *ash2*, *set1*, *shg1*, *spf1*, *swd1*, and *swd3* display increased mortality phenotypes in quiescence [11]. Hence, it is likely that the survival of Q cells requires upregulation of quiescence genes by Set1C.

## 4. Materials and Methods

### 4.1. Cell Culture

Fission yeast cells (Table 2) were grown in PMG (pombe minimal glutamate) media at 30 °C, 200 rpm until cells reached mid-log phase. Vegetative cells (V cells) were harvested at mid-log phase. For quiescence experiments, the mid-log phase cells were washed three times with PMG without nitrogen (PMG-N) media to completely remove the nitrogen, and then switched to PMG-N media and incubated for 24 h before harvesting the quiescent cells (Q cells).

### 4.2. RNA Extraction and qRT-PCR

As we know, 0.5–1 µg of input total RNA is recommended for real-time quantitative reverse transcription PCR (real-time qRT-PCR). Therefore, at least 125 × 10^6^ fission yeast cells (V cells and Q cells) were needed. The cells were collected by centrifugation at 3000× *g* for 5 min at 30 °C, followed by a single quick wash step at room temperature with 1 × PBS. After centrifugation, the cell pellets were snap-frozen on dry ice. The E.Z.N.A.^®^ Total RNA Kit I (OMEGA Bio-tek; Georgia, Norcross, GA, USA, R6834-02) was used for RNA extraction following the kit instructions with the following modifications for fission yeast cells: 500 µL lysis buffer from the Total RNA Kit (TRK) and 500 µL acid-washed glass beads were added into the cell pellets. The tubes were tightly closed and vortexed at 4 °C for 1 h to disrupt the cells. Subsequently, the tubes were centrifuged at 14,000× *g* for 5 min at 4 °C, and the cleared supernatant was transferred to a new tube. The remaining steps for RNA purifications were carried out according to the TRK kit instructions. For reverse transcription and qRT-PCR, we used commercial kits (LunaScript^®^ RT Supermix Kit) and SYBR green mix (KAPA SYBR^®^ FAST; Roche, Basel, Switzerland) according to the instructions, PCR primers (Table 3), and the Applied Biosystems 7500 Fast Real-Time PCR System apparatus.

### 4.3. Chromatin Immunoprecipitation Sequencing (ChIP-seq)

The Hu3112 strain was used for ChIP-seq. The mid-log phase vegetative cells (growing in PMG media) and quiescent cells (after switch to PMG-N media for 24 h) were cross-linked with a final concentration of 1% formaldehyde solution (Merck, Darmstadt, Germany, 252549) at 25 °C for 30 min and then quenched by a final concentration of 125 mM glycine at 25 °C for 5 min. The cross-linked cells were washed twice with cold 1 × PBS and snap-frozen using dry ice. The frozen cell pellets were thawed and re-washed with ChIP lysis buffer (50 mM HEPES-KOH (pH 7.5), 140 mM NaCl, 1 mM EDTA (pH 8.0), 1% (*w*/*v*) Triton X-100, 0.1% (*w*/*v*) SDS, and 0.1 sodium deoxycholate). After the final wash, the cell solution was transferred into pre-cooled Sarstedt screw-cap tubes containing 500 µL of 0.5 mm Zirconia/Silica Beads (Techtum, Nacka, Sweden, 11079105z). Cell lysis was performed using a FastPrep-24 machine (MP) until more than 90% of the cells were broken. After puncturing the bottom of the Sarstedt screw-cap tube and performing a low-speed centrifuge, the bottom part of the solution was collected, avoiding any beads. The solution was then transferred into 1.5 mL Bioruptor microtubes (Diagenode, Belgium, C30010016) and sonicated using a Bioruptor apparatus. After determining the chromatin concentration and fragment size, the ideal sheared chromatin (chromatin fragment sizes were around 500 base pairs) was stored at −80 °C.

For the immunoprecipitation step, the following antibodies were used: anti-Spike-in antibodies (61686, Active Motif, Sweden), anti-H3 antibodies (ab1791, Abcam, Cambridge, U.K.), anti-H3K9ac antibodies (ab4441, Abcam), anti-H3K4me3 antibodies (ab12209, Abcam), anti-H3K9me2 antibodies (ab1220, Abcam), anti-H3K9me3 antibodies (39062, Active Motif, Carlsbad, CA, USA), anti-RNA Pol II S2 antibodies (ab5095, Abcam), and anti-RNA Pol II S5 antibodies (ab5131, Abcam). The same amount of sheared chromatin, spike-in chromatin (53083, Active Motif), and final system volume were maintained for comparison.

After immunoprecipitation, three washing steps were performed using low-salt wash buffer (20 mM Tris-HCl (pH 8.0), 150 mM NaCl, 2 mM EDTA (pH 8.0), 1% (*w*/*v*) Triton X-100, and 0.1% SDS), high-salt wash buffer (20 mM Tris-HCl (pH 8.0), 500 mM NaCl, 2 mM EDTA (pH 8.0), 1% (*w*/*v*) Triton X-100, and 0.1% SDS), and LiCl wash buffer (10 mM Tris-HCl (pH 8.0), 250 mM LiCl, 1 mM EDTA (pH 8.0), 1% IGEPAL CA-630, and 0.1% sodium deoxycholate) successively. ChIP-DNA was extracted using the ChIP DNA Clean & Concentrator kit (ZYMO RESEARCH, D5205, Irvine, CA, USA), and DNA concentration was measured using the Qubit dsDNA HS assay kit (ThermoFisher, Frederick, MD, USA, Q33231). The sequencing library was prepared using the ThruPLEX DNA-Seq kit (TaKaRa, Shiga, Japan R400676) with the DNA HT Dual Index Kit—96N Set A (TaKaRa, R400660). Sequencing was performed using the Illumina Nextseq 2000 platform with the P3 v3 50 kit (36 + 8 + 8 + 36 cycles, single-end sequencing) at the BEA facility (Huddinge, Sweden).

### 4.4. Bioinformatics

Raw sequencing data from Nextseq 2000 (Bcl files) were converted and demultiplexed to fastq files using the bcl2fastq v2.20.0.422 program. The bwa 0.7.17 and Samtools 1.17 programs were used for alignment and file conversion. Later, we used deeptools 3.3.2 to compute pairwise Spearman correlation coefficients, normalize the bigwig files, and count the reads. The graphs were performed by using R. For volcano plots and bubble plots, batch effects were removed using DEseq2 [21]. Scripts will be made available in GitHub upon request.

## Figures and Tables

**Table 1:** Lists of genes changing their chromatin state in Q cells. The gene lists are derived from the intersections in Figure 6.

Systematic Gene Name	Gene Name/ GO Molecular Function	Chromatin State in Q Cells
		Active
SPAC1002.19	*urg1/ GTP cyclohydrolase*	Active
SPAC1039.10	*mmf2/ mitochondrial matrix protein*	Active
SPAC11D3.19		Active
SPAC2H10.01	*transcription factor*	Active
SPAC3C7.02c	*pil2/ meiotic eisosome*	Active
SPAC3G9.11c	*pdc201/ pyruvate decarboxylase*	Active
SPAC869.01	*hydrolase, cellular detoxification*	Active
SPAC869.03c	*urea transmembrane transporter*	Active
SPAC869.04	*formamidase, cellular detoxification*	Active
SPAC869.06c	*hry1/ cation binding protein*	Active
SPAC869.07c	*mel1/ alpha-galactosidase*	Active
SPAC869.08	*mel2/ isoaspartate methyltransferase*	Active
SPAC869.09		Active
SPAC9E9.17c		Active
SPAPB8E5.05	*mfm1/ M-factor precursor*	Active
SPAPJ691.02		Active
SPBC354.12	*gpd3/ glyceraldehyde dehydrogenase*	Active
SPBPB21E7.02c	*phosphoglycerate mutase*	Active
SPBPB21E7.10		Active
SPBPB21E7.11		Active
SPCC338.18		Active
SPCC417.02	*dad5/ DASH complex subunit*	Active
SPCC550.07	*fah2/ fatty-acid amide hydrolase*	Active
SPCC965.13	*transmembrane transporter*	Active
SPNCRNA.1415	*non-coding RNA*	Active
SPNCRNA.1530	*non-coding RNA*	Active
SPNCRNA.1670	*non-coding RNA*	Active
SPNCRNA.352	*non-coding RNA*	Active
SPNCRNA.51	*non-coding RNA*	Active
SPNCRNA.577	*non-coding RNA*	Active
SPNCRNA.607	*non-coding RNA*	Active
SPNCRNA.877	*non-coding RNA*	Active
SPNCRNA.987	*non-coding RNA*	Active
		
SPAC1834.04	*hht1/ histone H3*	Repressed
SPAC30D11.11	*izh3/ ER membrane protein*	Repressed
SPAC31G5.04	*lys12/ homoisocitrate dehydrogenase*	Repressed
SPBC1711.07	*rrb1/ chaperone for ribosomal protein*	Repressed
SPCC74.05	*rpl2702/ 60S ribosomal protein*	Repressed
SPNCRNA.1461	*non-coding RNA*	Repressed
SPNCRNA.979	*non-coding RNA*	Repressed
		

**Table 2:** List of strains.

Strain Number	Genotype	Reference
Hu3112	*mat1-M smt-0 pht1-myc*	[12]
Hu2909	*mat1-M smt-0*	[11]
Hu3119	*mat1-M smt-0 set1Δ::kanMX*	[11]

**Table 3:** List of PCR primers.

Gene	Forward	Reverse
SPAC869.09	GCCTGATCCTGCACATATTATCG	TTTGCATGACGCCTACCTTTCTG
mel1	GTTACAAGCGAATGTCTGATGCTC	ATACTCCTCAAAGCTCATACCTCC
SPAC869.06c	GTCGGCAAACGACTATGATACTG	AATATCATGTTGCTCCTCCTCGTC
SPAC869.04	AAATAAACCTGCATGGGAGCAACC	AATCCTCCTCCATTCTCTTTCG
SPAC869.03c	ACCAGGGATATGGATATGGAATCG	CACATACTTCCACATACGCCATAC
SPBPB21E7.02c	GCCCTTCATAATTGGTTGGTTGAC	GTTCCGTATAATCCTCGTCTCAGTG
SPBPB21E7.10	GTTGAAAGGAGAAGTCGAAGAAGG	CTATTCGCTCTCCTTTCTTTCACC
SPBPB21E7.11	TGTTATGCGTGCGTTCTTCATCC	TGATTGAGTATCAGGTGCGCTTG
gmh2	CTTGATTGCTGGCGCGTTTCTATAC	TTTGCTGAGGCTCTGACTCGTATG
SPRRNA.47(rRNA)	TAGCCAAATGCCTCGTCATC	CATACTCCCACTTATCCTACACCC

## Data Availability

The ChIP-seq data have been submitted to the Gene Expression Omnibus under the accession number GSE280066.

## Supplementary Materials

The following supporting information can be downloaded at: https://www.mdpi.com/article/10.3390/epigenomes8040039/s1, Figure S1: Volcano plot of histone modification changes in ORF regions in Q vs. V cells; Figures S2 and S3: Pie charts showing analysis of histone modification changes in quiescence genes in TSS and ORF regions, respectively. Figure S4: Bar diagrams showing additional repeats of qRT-PCR analysis of expression of core quiescence genes in Q vs. V cells. Figure S5: quantitative RT-PCR analysis of core quiescence genes. Supplementary Table S1 is an excel file with details of epigenome changes in early genes and core quiescence genes.

## References

[1] de Morree A., Rando T.A. (2023). Regulation of adult stem cell quiescence and its functions in the maintenance of tissue integrity. Nat. Rev. Mol. Cell Biol..

[2] Roche B., Arcangioli B., Martienssen R. (2017). Transcriptional reprogramming in cellular quiescence. RNA Biol..

[3] Li Y., Wang Z., Ajani J.A., Song S. (2021). Drug resistance and Cancer stem cells. Cell Commun. Signal.

[4] Hayles J., Nurse P. (2018). Introduction to Fission Yeast as a Model System. Cold Spring Harb. Protoc..

[5] Sajiki K., Hatanaka M., Nakamura T., Takeda K., Shimanuki M., Yoshida T., Hanyu Y., Hayashi T., Nakaseko Y., Yanagida M. (2009). Genetic control of cellular quiescence in *S. pombe*. J. Cell Sci..

[6] Yanagida M. (2009). Cellular quiescence: Are controlling genes conserved?. Trends Cell Biol..

[7] Allshire R.C., Ekwall K. (2015). Epigenetic Regulation of Chromatin States in Schizosaccharomyces pombe. Cold Spring Harb. Perspect. Biol..

[8] Bonitto K., Sarathy K., Atai K., Mitra M., Coller H.A. (2021). Is There a Histone Code for Cellular Quiescence?. Front. Cell Dev. Biol..

[9] Roche B., Arcangioli B., Martienssen R.A. (2016). RNA interference is essential for cellular quiescence. Science.

[10] Joh R.I., Khanduja J.S., Calvo I.A., Mistry M., Palmieri C.M., Savol A.J., Sui S.J., Sadreyev R.I., Aryee M.J., Motamedi M. (2016). Survival in Quiescence Requires the Euchromatic Deployment of Clr4/SUV39H by Argonaute-Associated Small RNAs. Mol. Cell.

[11] Zahedi Y., Durand-Dubief M., Ekwall K. (2020). High-Throughput Flow Cytometry Combined with Genetic Analysis Brings New Insights into the Understanding of Chromatin Regulation of Cellular Quiescence. Int. J. Mol. Sci..

[12] Zahedi Y., Zeng S., Ekwall K. (2023). An essential role for the Ino80 chromatin remodeling complex in regulation of gene expression during cellular quiescence. Chromosome Res..

[13] Dehé P.-M., Dichtl B., Schaft D., Roguev A., Pamblanco M., Lebrun R., Rodríguez-Gil A., Mkandawire M., Landsberg K., Shevchenko A. (2006). Protein interactions within the Set1 complex and their roles in the regulation of histone 3 lysine 4 methylation. J. Biol. Chem..

[14] Marguerat S., Schmidt A., Codlin S., Chen W., Aebersold R., Bähler J. (2012). Quantitative analysis of fission yeast transcriptomes and proteomes in proliferating and quiescent cells. Cell.

[15] Young C.P., Hillyer C., Hokamp K., Fitzpatrick D.J., Konstantinov N.K., Welty J.S., Ness S.A., Werner-Washburne M., Fleming A.B., Osley M.A. (2017). Distinct histone methylation and transcription profiles are established during the development of cellular quiescence in yeast. BMC Genom..

[16] Lee J., Kang S., Lilja K.C., Colletier K.J., Scheitz C.J., Zhang Y.V., Tumbar T. (2016). Signalling couples hair follicle stem cell quiescence with reduced histone H3 K4/K9/K27me3 for proper tissue homeostasis. Nat. Commun..

[17] Smith E., Shilatifard A. (2013). Transcriptional elongation checkpoint control in development and disease. Genes Dev..

[18] Wang H., Fan Z., Shliaha P.V., Miele M., Hendrickson R.C., Jiang X., Helin K. (2023). H3K4me3 regulates RNA polymerase II promoter-proximal pause-release. Nature.

[19] Larochelle M., Robert M.-A., Hébert J.-N., Liu X., Matteau D., Rodrigue S., Tian B., Jacques P., Bachand F. (2018). Common mechanism of transcription termination at coding and noncoding RNA genes in fission yeast. Nat. Commun..

[20] Roguev A., Schaft D., Shevchenko A., Pijnappel W., Wilm M., Aasland R., Stewart A. (2001). The Saccharomyces cerevisiae Set1 complex includes an Ash2 homologue and methylates histone 3 lysine 4. EMBO J..

[21] Love M.I., Huber W., Anders S. (2014). Moderated estimation of fold change and dispersion for RNA-seq data with DESeq2. Genome Biol..

