# Supplementary material for: Epigenome Mapping in Quiescent Cells Reveals a Key Role for H3K4me3 in Regulation of RNA Polymerase II Activity
Source: Epigenomes. 2024 Oct 22;8(4):39. doi: 10.3390/epigenomes8040039 (PMC11503321; doi:10.3390/epigenomes8040039)
Supplement: Supplementary file 1 [file epigenomes-08-00039-s001.zip › Supplementary FIgures Zeng et al Epigenomes_revised.pptx]

## Slide 1
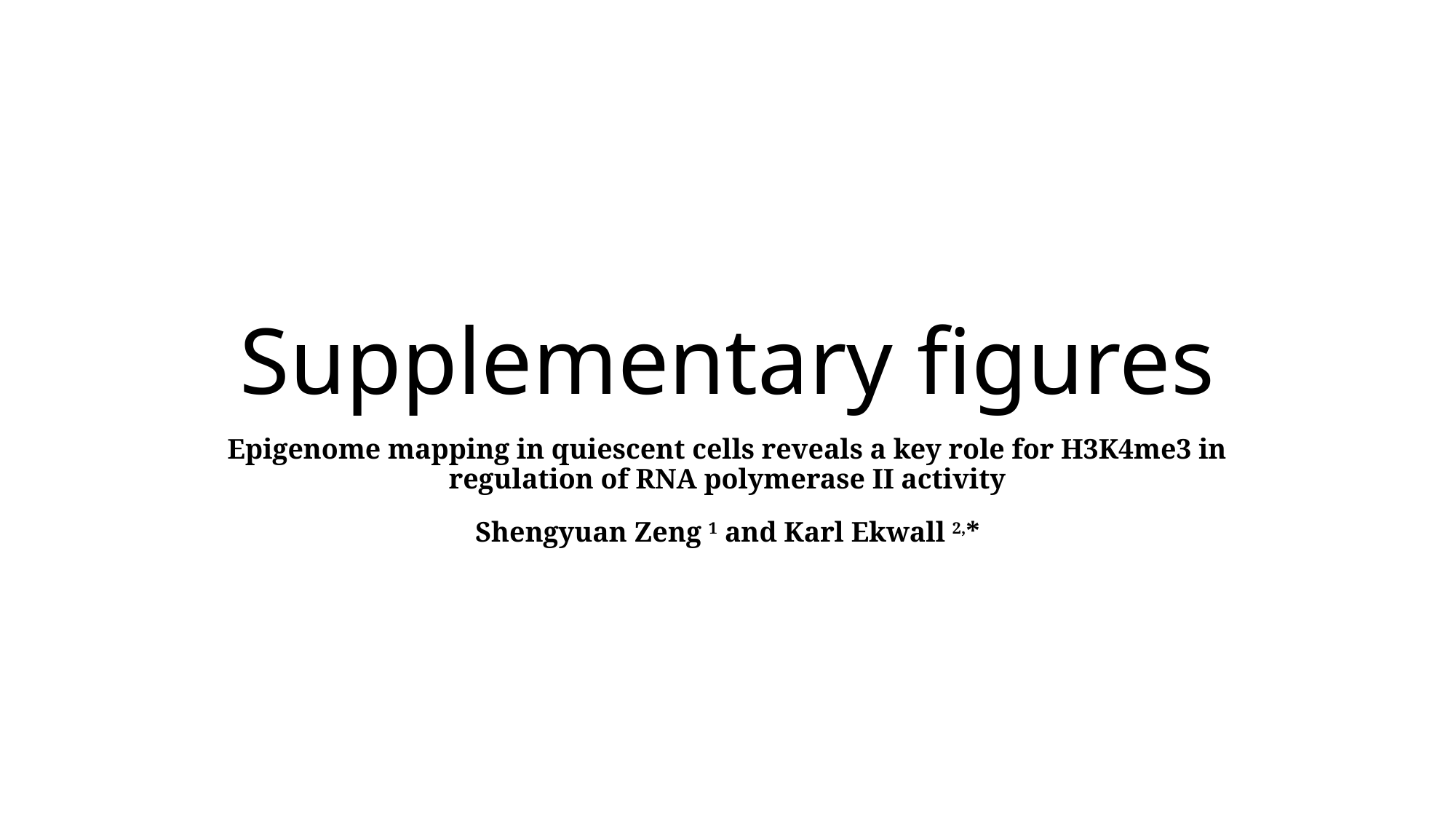

# Supplementary figures
Epigenome mapping in quiescent cells reveals a key role for H3K4me3 in regulation of RNA polymerase II activity
Shengyuan Zeng 1 and Karl Ekwall 2,*

## Slide 2
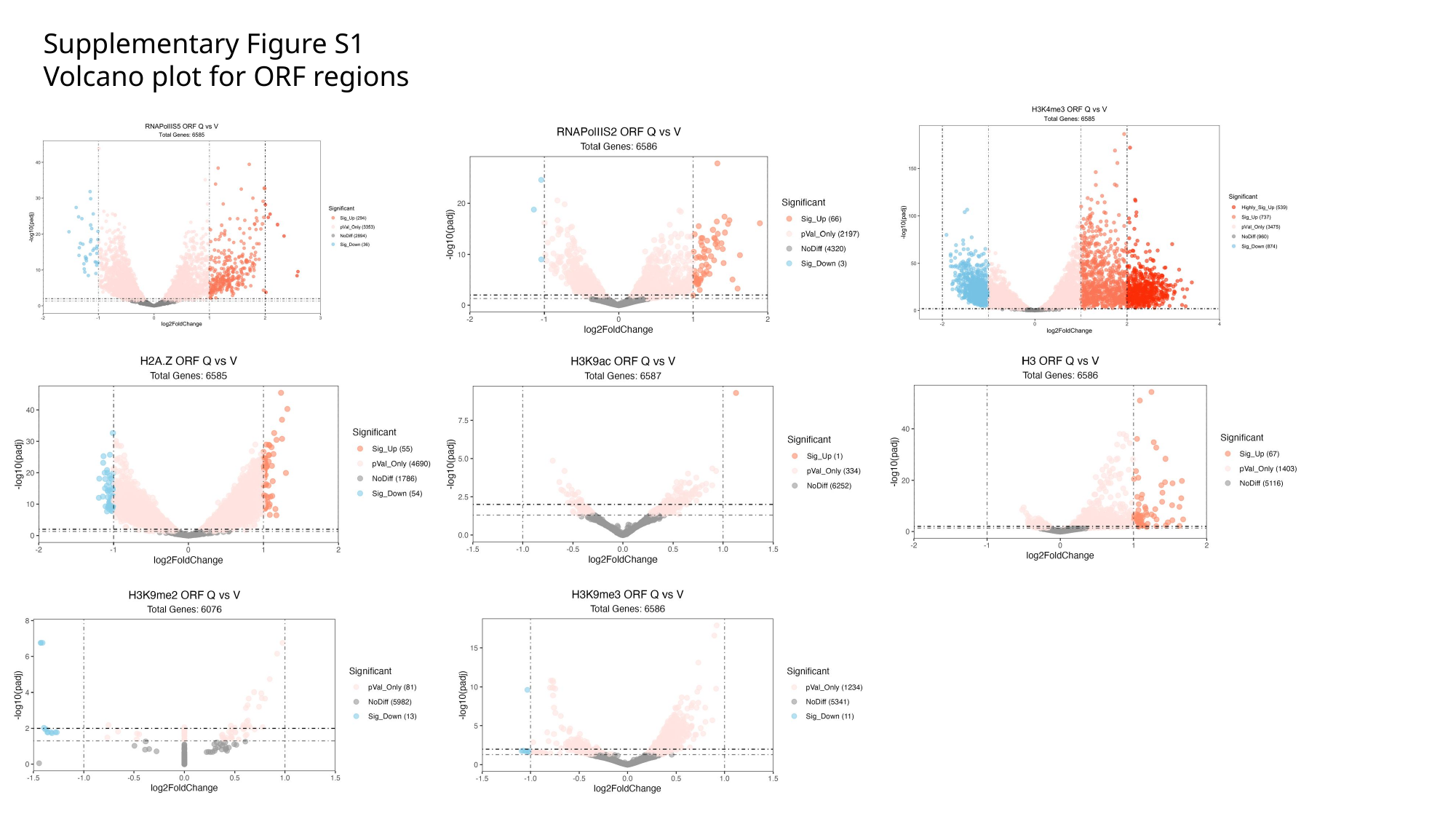

Supplementary Figure S1
Volcano plot for ORF regions

## Slide 3
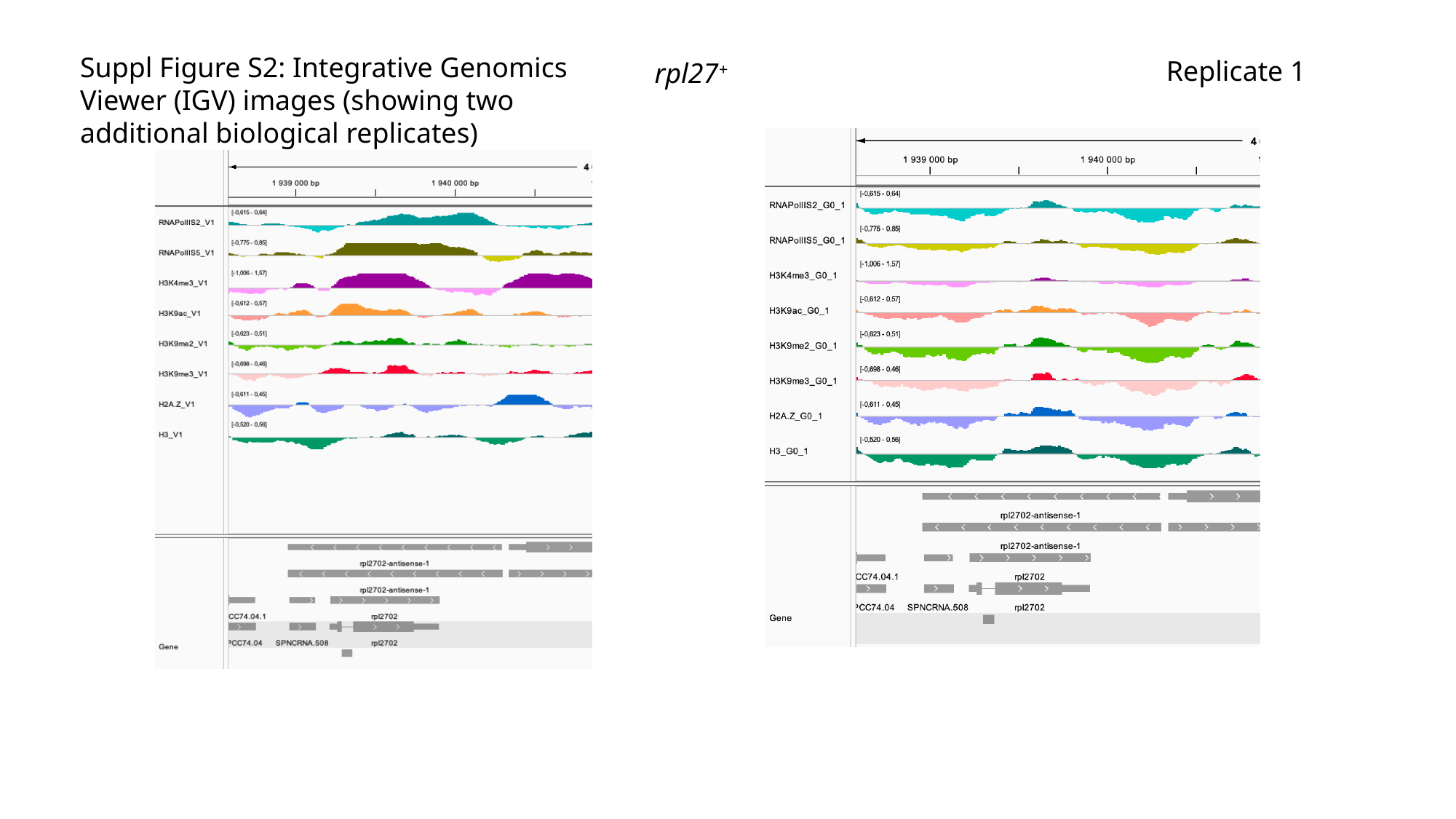

Suppl Figure S2: Integrative Genomics Viewer (IGV) images (showing two additional biological replicates)
Replicate 1
rpl27+

## Slide 4
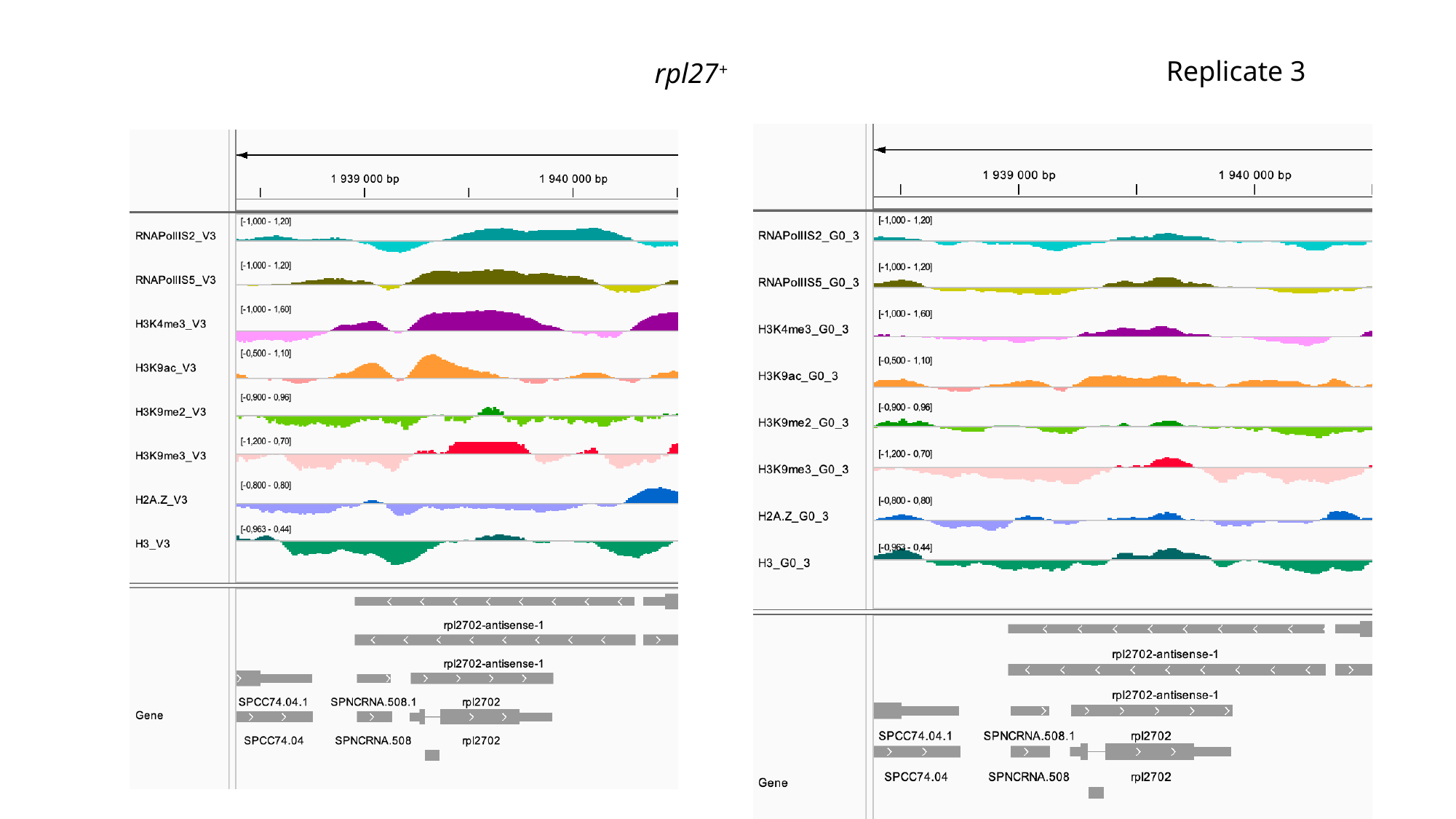

Replicate 3
rpl27+

## Slide 5
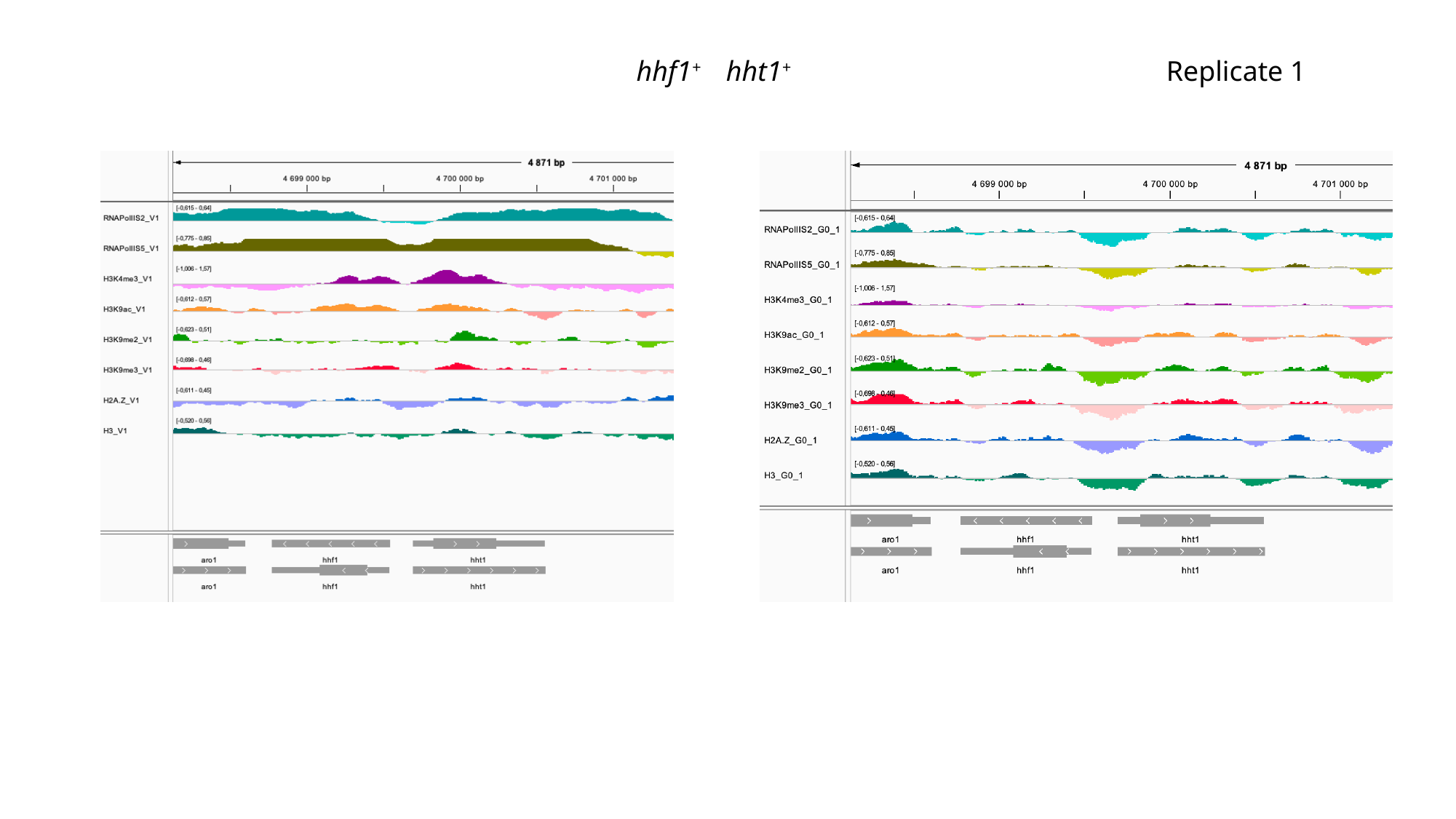

hhf1+ hht1+
Replicate 1

## Slide 6
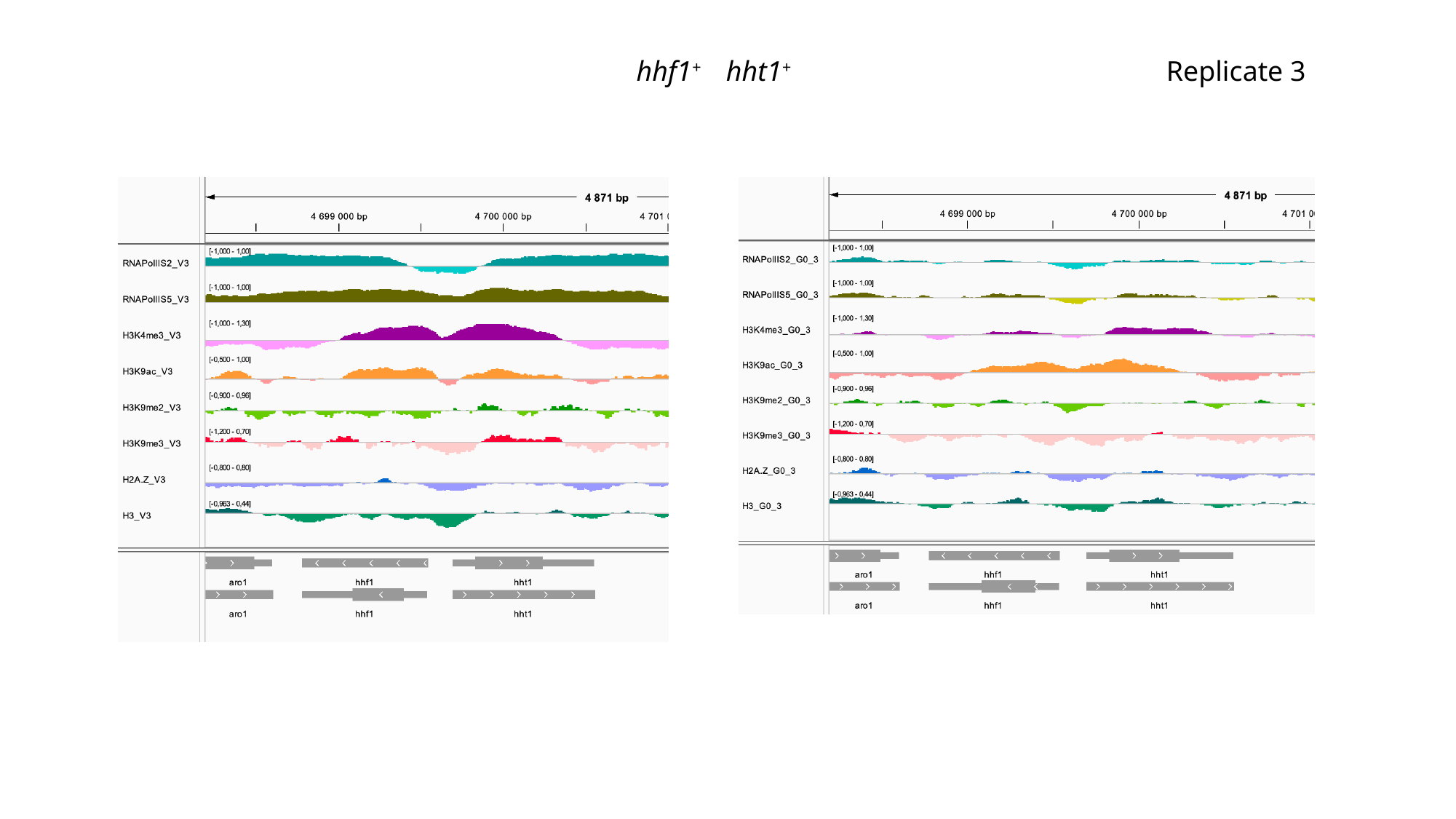

hhf1+ hht1+
Replicate 3

## Slide 7
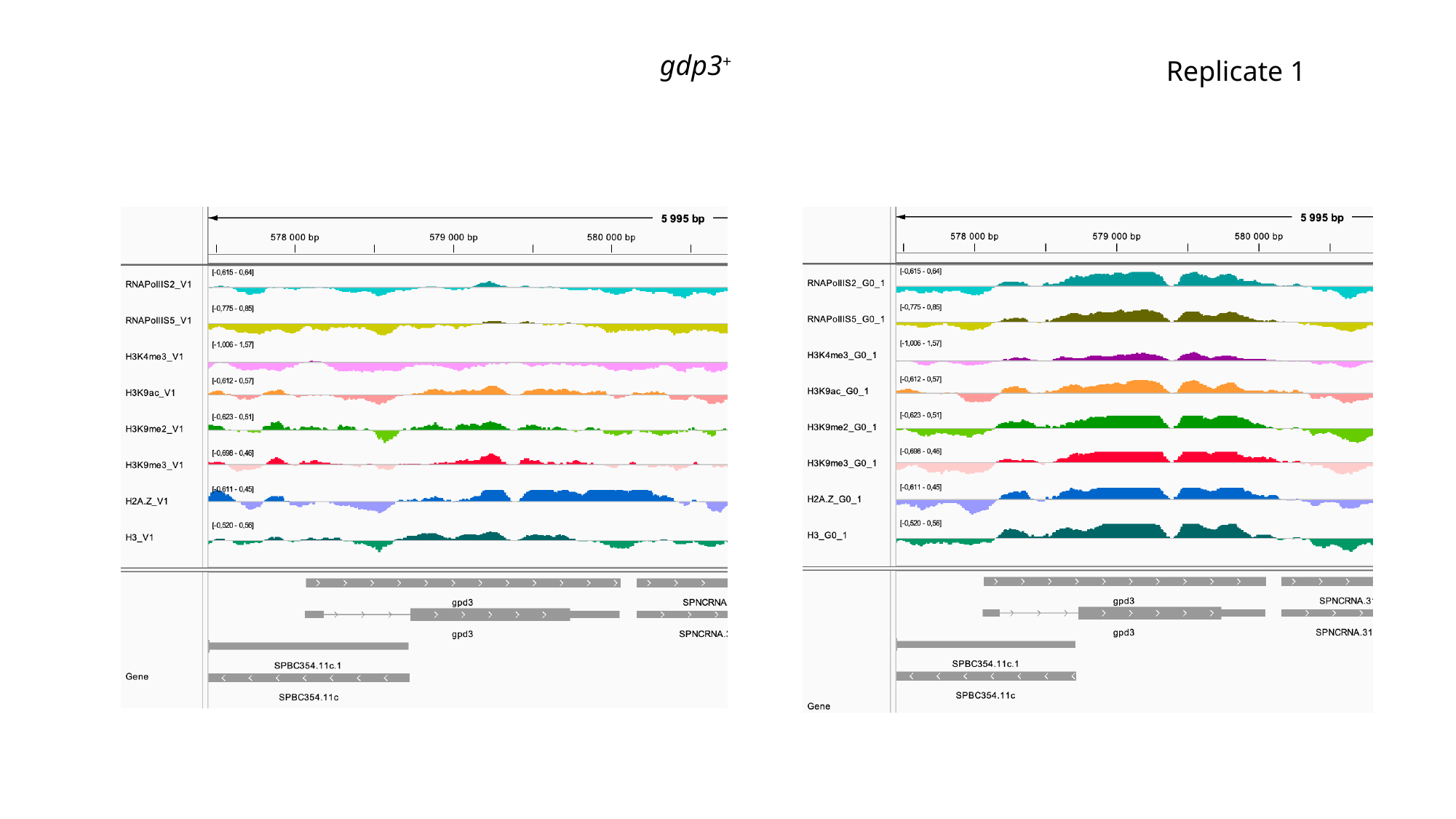

gdp3+
Replicate 1

## Slide 8
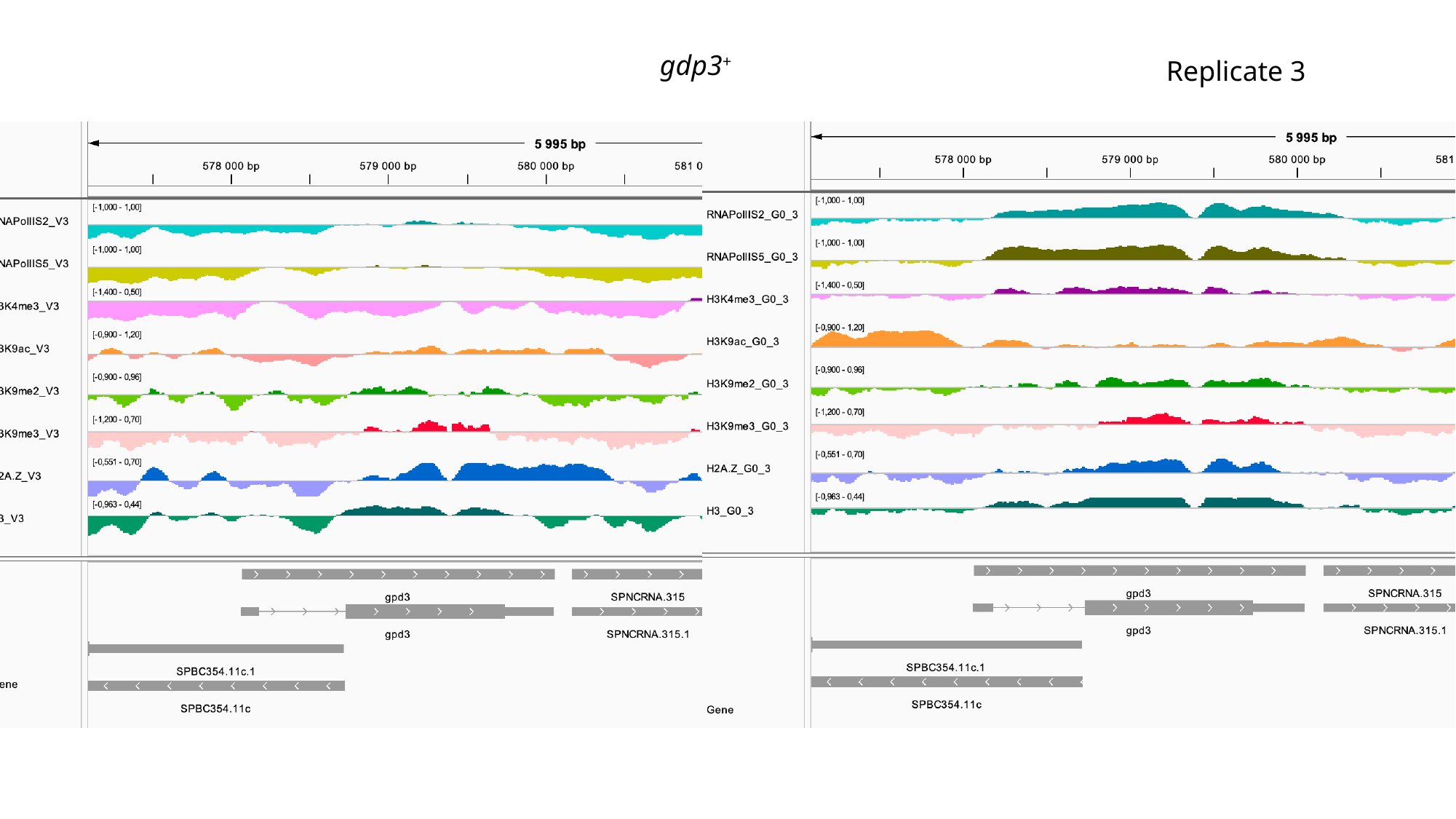

gdp3+
Replicate 3

## Slide 9
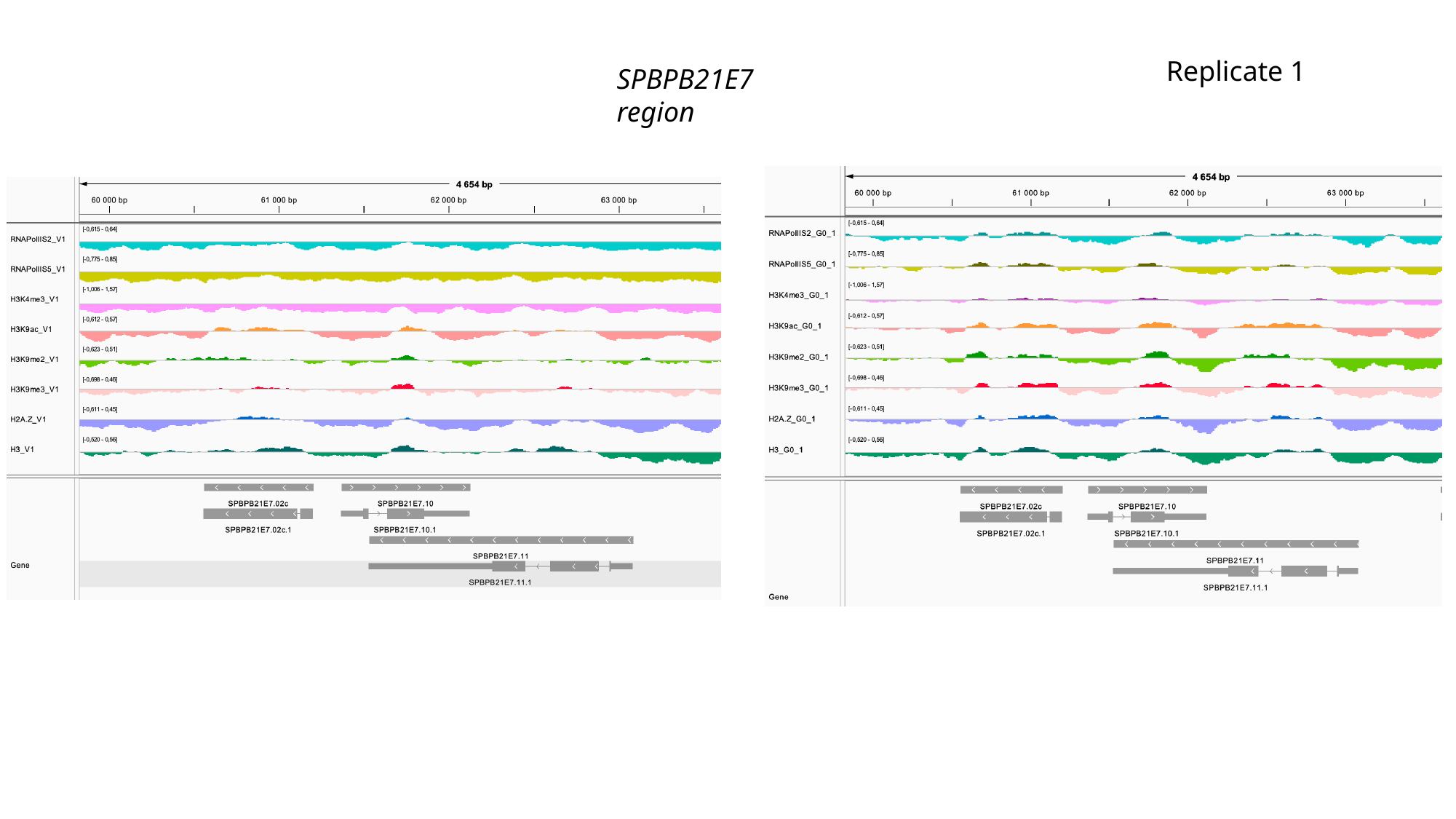

Replicate 1
SPBPB21E7 region

## Slide 10
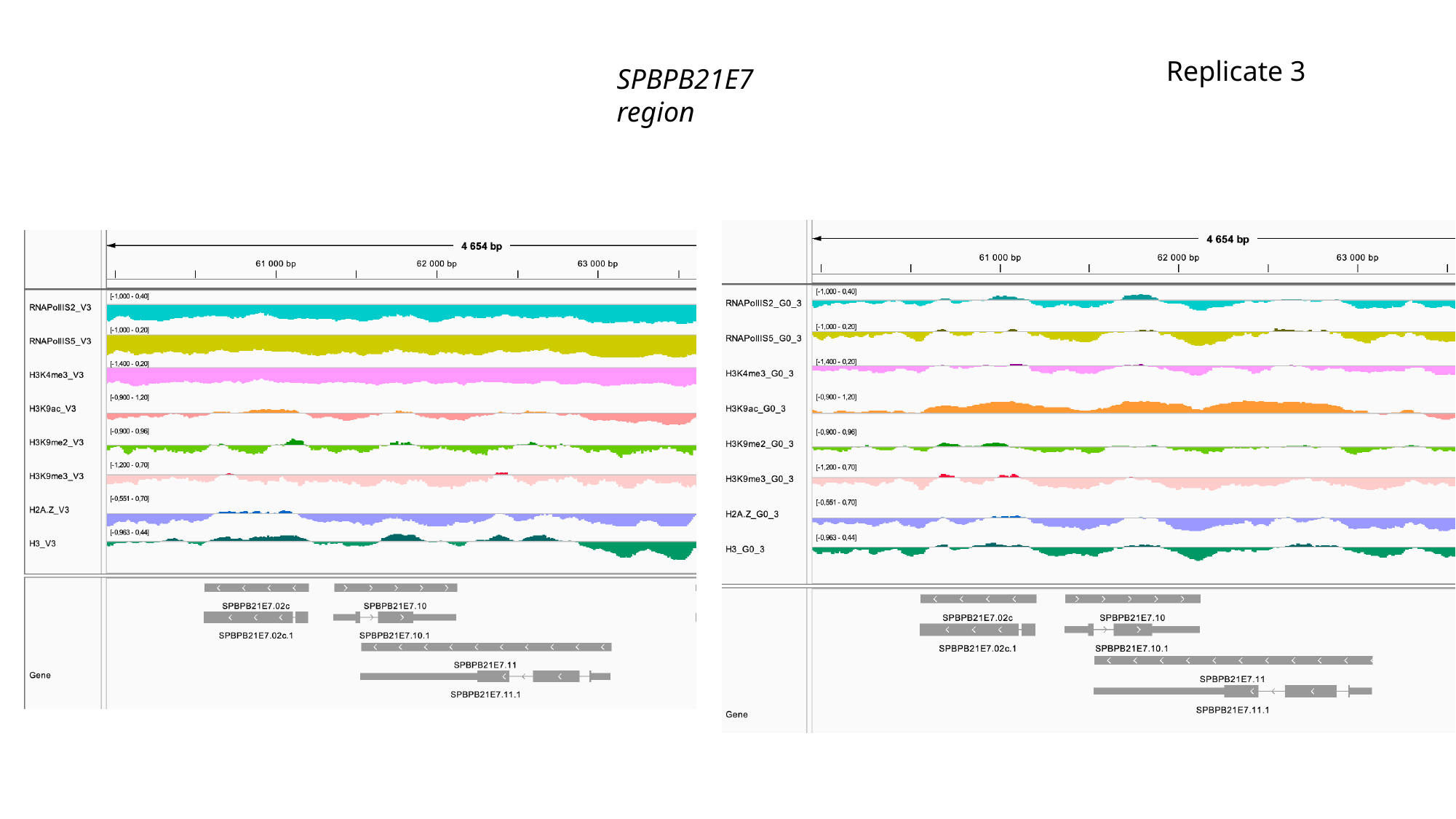

Replicate 3
SPBPB21E7 region

## Slide 11
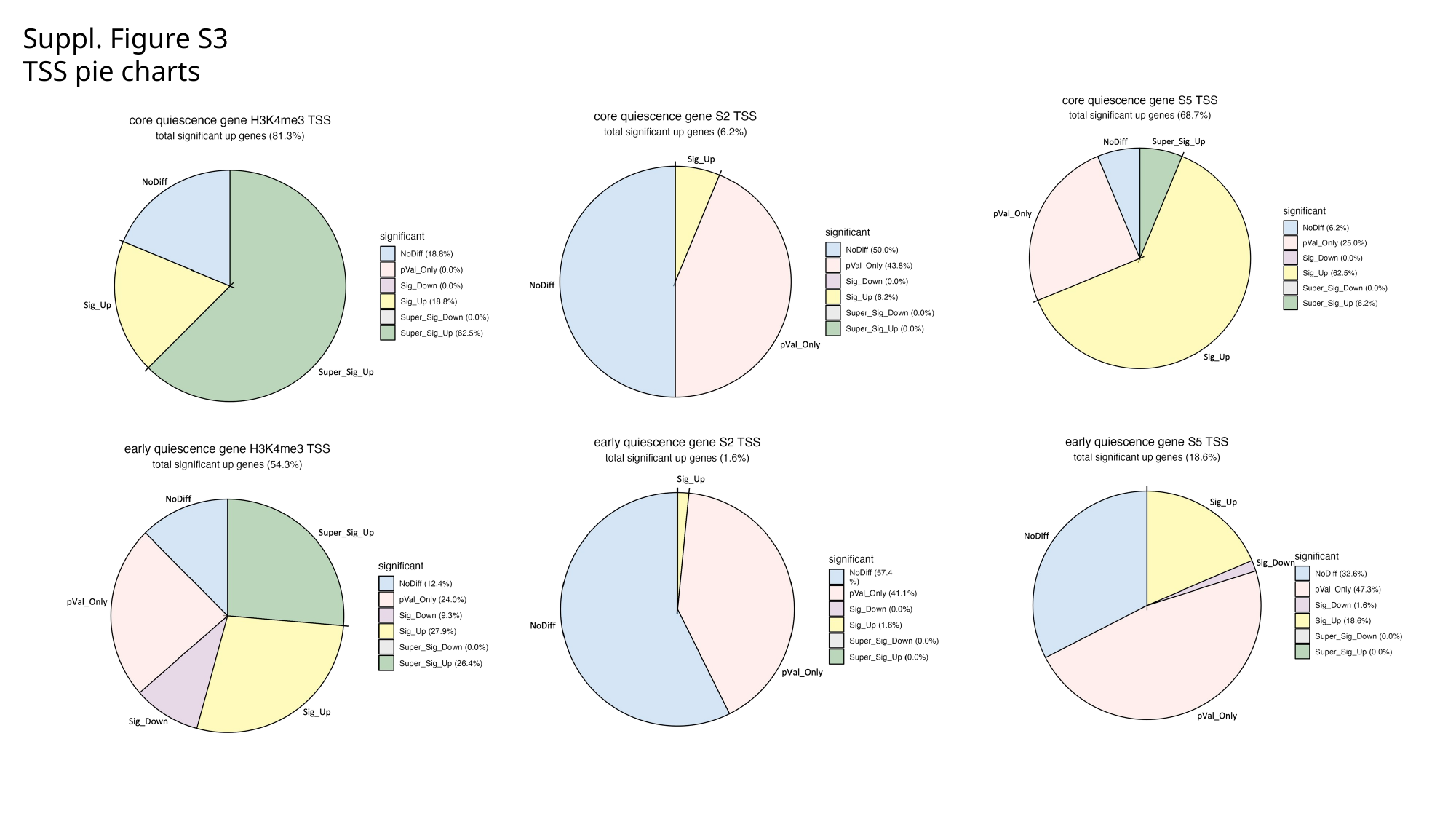

Suppl. Figure S3
TSS pie charts

## Slide 12
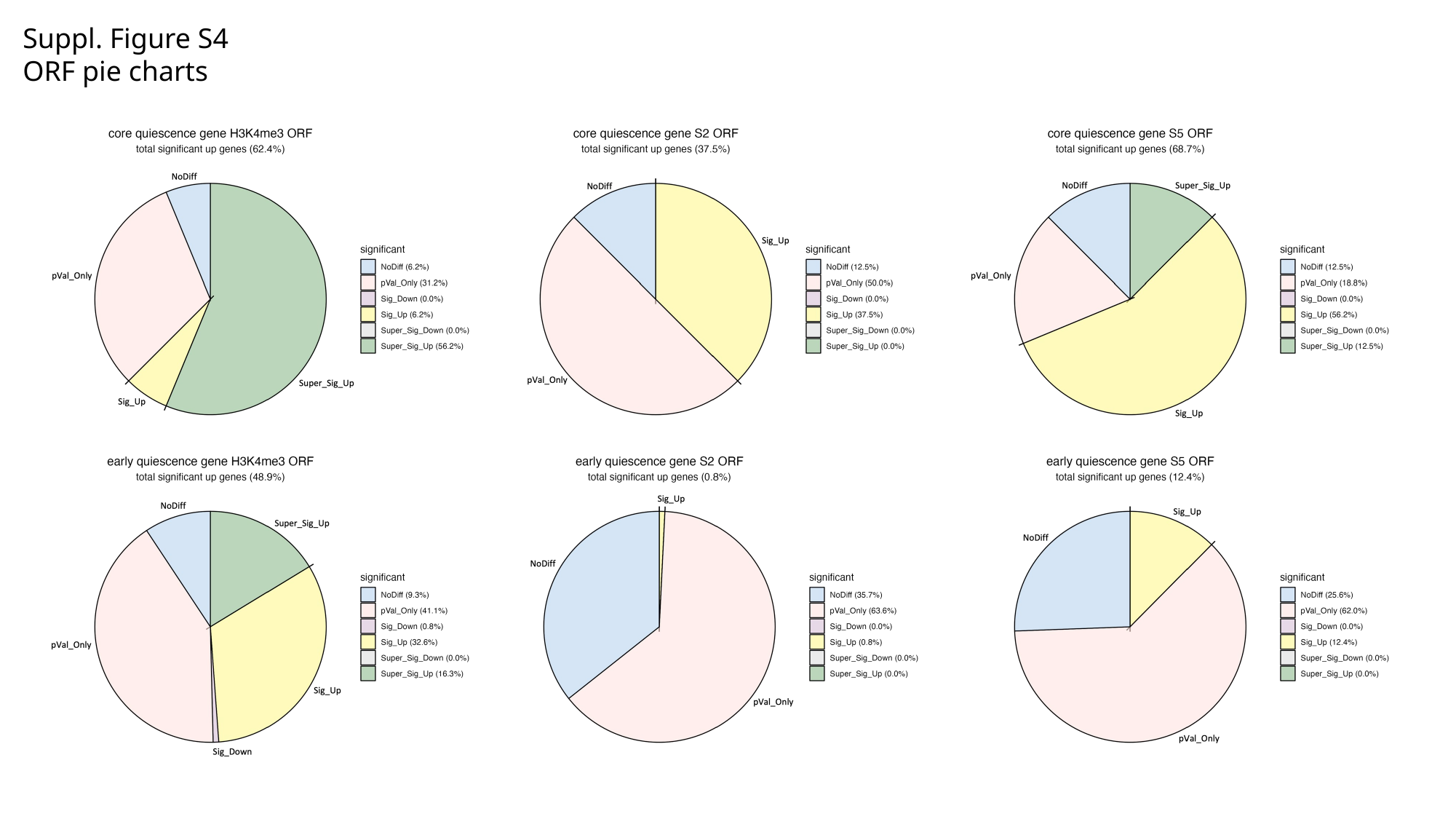

Suppl. Figure S4
ORF pie charts

## Slide 13
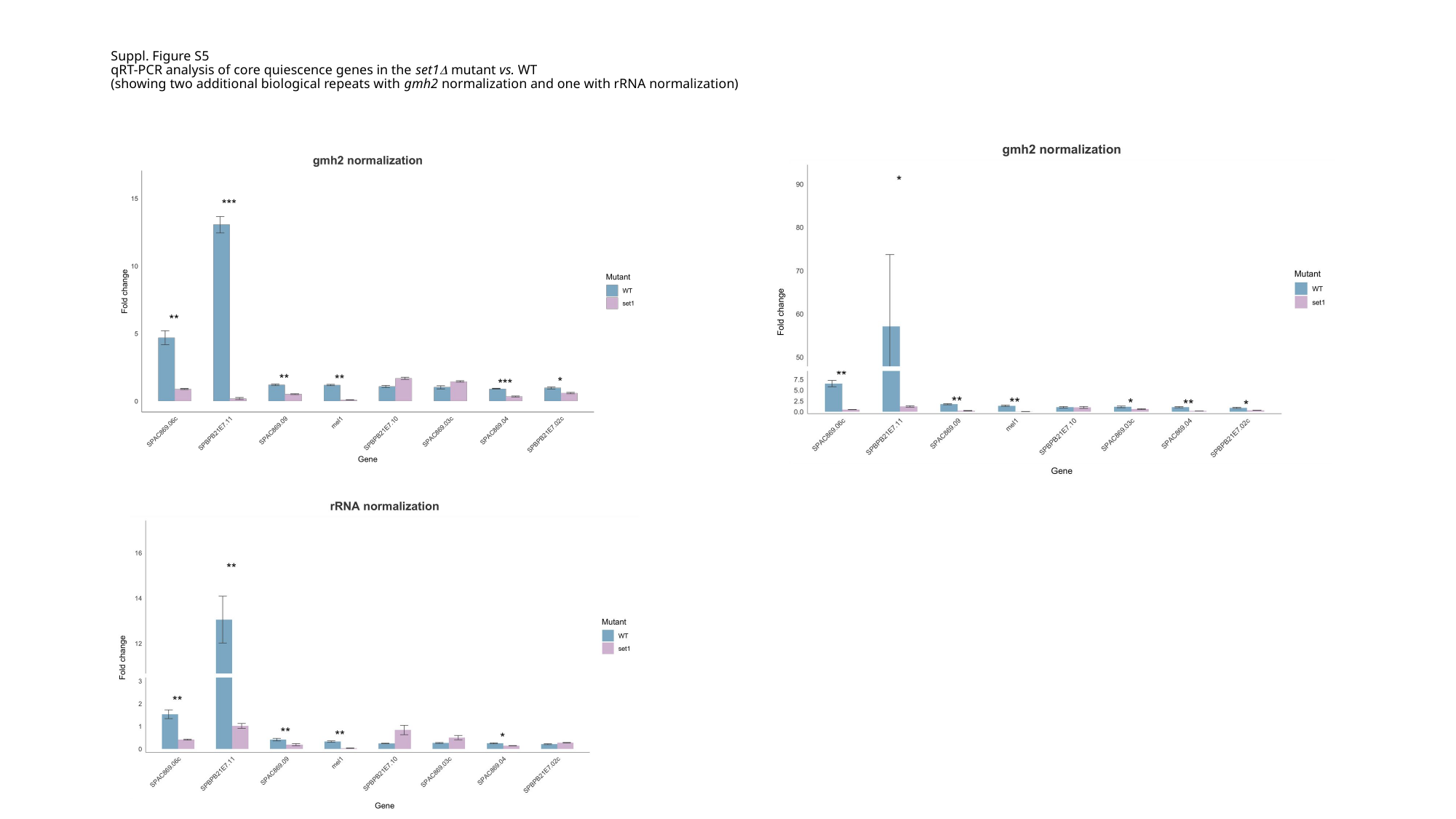

# Suppl. Figure S5 qRT-PCR analysis of core quiescence genes in the set1D mutant vs. WT (showing two additional biological repeats with gmh2 normalization and one with rRNA normalization)
